# Counting the genetic ancestors from source populations in members of an admixed population

**DOI:** 10.1093/genetics/iyae011

**Published:** 2024-01-30

**Authors:** Lily Agranat-Tamir, Jazlyn A Mooney, Noah A Rosenberg

**Affiliations:** Department of Biology, Stanford University, Stanford, CA 94305, USA; Department of Quantitative & Computational Biology, University of Southern California, Los Angeles, CA 90089, USA; Department of Biology, Stanford University, Stanford, CA 94305, USA

**Keywords:** admixture, African-American population, genealogies, genetic ancestors, pedigrees

## Abstract

In a genetically admixed population, admixed individuals possess genealogical and genetic ancestry from multiple source groups. Under a mechanistic model of admixture, we study the number of distinct ancestors from the source populations that the admixture represents. Combining a mechanistic admixture model with a recombination model that describes the probability that a genealogical ancestor is a genetic ancestor, for a member of a genetically admixed population, we count *genetic* ancestors from the source populations—those genealogical ancestors from the source populations who contribute to the genome of the modern admixed individual. We compare patterns in the numbers of genealogical and genetic ancestors across the generations. To illustrate the enumeration of genetic ancestors from source populations in an admixed group, we apply the model to the African-American population, extending recent results on the numbers of African and European genealogical ancestors that contribute to the pedigree of an African-American chosen at random, so that we also evaluate the numbers of African and European *genetic* ancestors who contribute to random African-American genomes. The model suggests that the autosomal genome of a random African-American born in the interval 1960–1965 contains genetic contributions from a mean of 162 African (standard deviation 47, interquartile range 127–192) and 32 European ancestors (standard deviation 14, interquartile range 21–43). The enumeration of genetic ancestors can potentially be performed in other diploid species in which admixture and recombination models can be specified.

## Introduction

The genealogical pedigree of any individual person can be viewed as a structure that has been shaped by demographic events such as migrations and population admixtures. The pedigree contains the individual’s recent ancestors, who have contributed in a genealogical sense to the individual, and with increasing probability as time proceeds toward the most recent generations, in a genetic sense as well.

The distinction between genealogical and genetic ancestry is inconsequential in recent generations: an individual necessarily contains genetic material from both parents, and almost certainly from all 4 grandparents and 8 great-grandparents as well. However, genetic transmission involves chromosomal segments, the number of which is finite. Hence, going back in time, the number of genealogical ancestors increases rapidly, and proportionally fewer of them are genetic ancestors: individuals who contribute to the genetic material of the modern individual. In the memorable description of Donnelly (1983), “This means that someone descended from the Scottish poet Robert Burns (born 1759) probably carries some of his genes, but that someone unilineally descended from the English playwright William Shakespeare (born 1564) is unlikely to have any genes in common with him.”

A number of studies have explored the peculiar consequences of the distinction between genealogical and genetic ancestors (Wiuf and Hein 1997; Baird *et al.* 2003; Matsen and Evans 2008; Gravel and Steel 2015; Buffalo *et al.* 2016; Kelleher *et al.* 2016). For example, one simulation study (Rohde *et al.* 2004), based on earlier mathematical work (Chang 1999), argued that the most recent genealogical ancestor shared by all living humans might have lived as few as 5,000 years ago, even though the most recent *genetic* ancestor lived much earlier. The rate at which recent genealogical ancestors dissipate from an individual’s genetic ancestry has been studied by Coop (2013), who used approximations to the human recombination process in order to calculate the number of autosomal fragments a genealogical ancestor passes to a descendant. Through that quantity, Coop (2013) computed the probability that a genealogical ancestor *k* generations ago is also a genetic ancestor. This analysis finds that although the number of genealogical ancestors grows exponentially in the number of generations back from the present, the number of genetic ancestors grows only linearly.

Recent admixture introduces a new dimension to the challenge of understanding the distinction between genealogical and genetic ancestry. In a recently admixed population, genealogical ancestors ultimately trace to 2 or more source populations. Some of these genealogical ancestors are genetic ancestors and some are not, so that the fraction of the genetic ancestors that trace to a specific source group need not equal the corresponding fraction of the genealogical ancestors that trace to that source.

Building on a mechanistic admixture model (Verdu and Rosenberg 2011), we have devised a model for counting genealogical ancestors in an admixed individual’s pedigree (Mooney *et al.* 2023), evaluating the numbers of individuals that enter the pedigree from each specific source population. Our goal here is to extend this genealogical model of an admixed pedigree to count the *genetic* ancestors that enter the pedigree. That is, we seek to count genetic ancestors from a certain source population that contribute to an individual’s genome, considering genetic ancestors in each generation in the pedigree.

To answer the new question posed by the study—*how many genetic ancestors from the source populations does the genetic admixture of a random member of an admixed population represent?*—we combine 2 mathematical approaches. The first is the extension of the admixture model studied by Mooney *et al.* (2023). The second is the method of Coop (2013) for approximating the probability that a genealogical ancestor is also a genetic ancestor. We develop a model that counts across the generations both genealogical and genetic ancestors from a certain source population of an admixed individual. We apply it to the African-American population, elaborating on the strictly genealogical approach of Mooney *et al.* (2023).

For this purpose, extending the work of Mooney *et al.* (2023), for a member of the admixed population, we study the random number of admixed *genealogical* ancestors in the pedigree in each generation by proceeding recursively back in time. From this random variable, we evaluate properties of the number of *genetic* ancestors from the admixed population and the number of genetic ancestors from the *source* populations, as well as the number of genealogical ancestors from the source populations as studied by Mooney *et al.* (2023).

## The model

### Admixture process

We build upon the model of Verdu and Rosenberg (2011) and Mooney *et al.* (2023), which considers the formation of a new admixed population. Two source populations that were present in generation 0 form the new admixed population in generation 1. After the initial admixture event, in each subsequent generation after generation 1, individuals from both source populations and the admixed population can be parents of an individual in the admixed population. Our interest is in an admixed individual in generation *g* after the initial admixture.

**Fig. 1.**
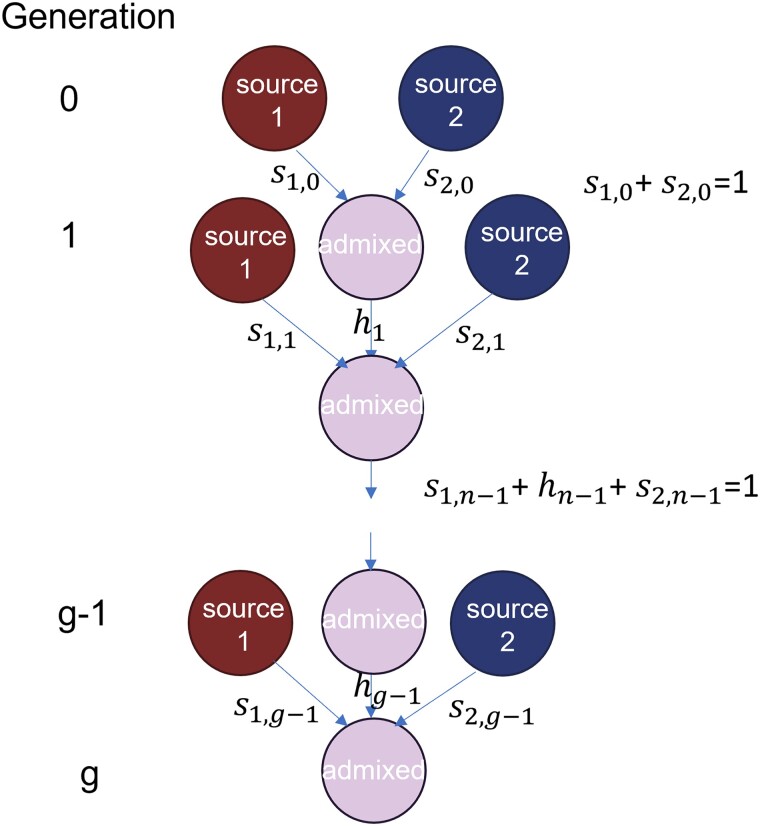
The general admixture model. Starting from generation 0, 2 source populations form an admixed population in generation 1, with admixture proportions $s_{1,0}$ and $s_{2,0}$. In the following generations, n=2,3,…,g, the admixed population receives contributions from both the source populations and the admixed population, in proportions $s_{1,n-1}$, $s_{2,n-1}$, and $h_{n-1}$.

We call the source populations “source 1” and “source 2.” For each n=1,2,…,g, we denote by $s_{1,n-1}$ the probability that for an admixed individual in generation *n* (*n* generations after members of generation 0 admix to form generation 1), a specific parent is from source population 1. We denote by $h_{n-1}$ the probability that the parent is from the admixed population, and by $s_{2,n-1}$ the corresponding probability for source 2. Therefore, for each n=1,2,…,g, we have $s_{1,n-1}+h_{n-1}+s_{2,n-1}=1$, recalling that $h_{0}=0$ (Fig. 1). The 2 parents are independent and identically distributed, amounting to an assumption that they are exchangeable members of the previous generation. The population is large, so that the chance that a particular individual is sampled twice can be ignored.

### Genealogical ancestors in a pedigree

**Fig. 2.**
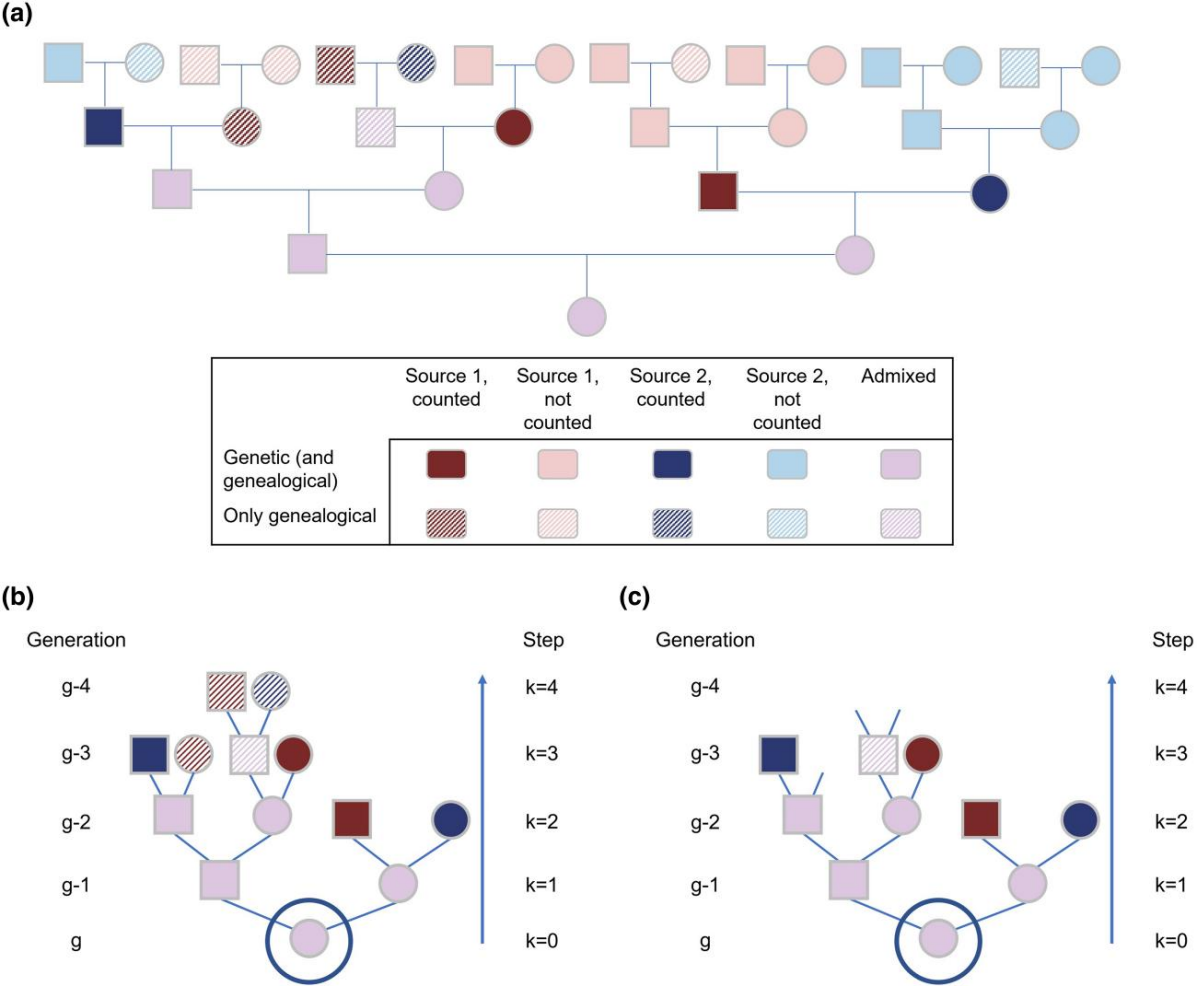
Counting genealogical and genetic ancestors from the source populations for an admixed individual. a) Pedigree of an admixed individual. Ancestors can be from source populations or from the admixed population itself. Ancestors from the source populations can be both genealogical *and* genetic ancestors (solid color), or genealogical ancestors only (striped). Along each genealogical line that reaches a source population, we count the most recent ancestor (dark color). b) Counting genealogical ancestors from source populations. For the pedigree in a), this panel goes back in time from an admixed individual in generation *g* (circled), on each line stopping when a source population is reached. The number of individuals from source 1 is 4 (red), and the number from source 2 is 3 (blue). c) Counting *genetic* ancestors from source populations. As in b), we traverse all admixed individuals in the pedigree, irrespective of genetic ancestry status. However, if a source-1 or source-2 ancestor is not a genetic ancestor, then that individual is not tabulated. Note that for ease of interpretation, the figure contains a higher number of genealogical but nongenetic ancestors than is likely in real pedigrees.

Consider Fig. 2a, describing the pedigree of an admixed individual. Tracing back from the admixed individual on each genealogical line, we eventually reach genealogical ancestors from the source populations. In each lineage that reaches ancestors who are only in source populations, we tabulate only the most recent one in our count of genealogical ancestors from source populations.

In the figure, some genealogical ancestors are genetic ancestors and some are not. In Mooney *et al.* (2023), we counted genealogical ancestors; the mathematical strategy followed previous studies (Verdu and Rosenberg 2011; Goldberg *et al.* 2014; Goldberg and Rosenberg 2015; Goldberg *et al.* 2020; Kim *et al.* 2021), in which source ancestry proportions were calculated recursively, beginning with the count of ancestors one generation after the initial admixture (n=1), and moving forward in time.

To count genetic ancestors, the approach of Mooney *et al.* (2023) is not straightforward to apply, because the probability that a genealogical ancestor is a genetic ancestor depends on that ancestor’s number of generations back from the present, even if the admixture process itself is constant in time. Further, a genetic ancestor of an individual in some generation g−n, with 0<n≤g, is not necessarily a genetic ancestor of the individual of interest in generation *g*.

To address these problems, we develop a model in which we count genealogical and genetic ancestors by proceeding backward in time (Fig. 2b and c). Tracing back from the admixed individual of interest in generation *g*, we examine, in each step, the parents of all the admixed individuals present in the pedigree. We tabulate those who are from a certain source population in our count of genealogical ancestors from that source population (Fig. 2b). We tabulate as genetic ancestors those who, in addition to being genealogical ancestors from the source, are also genetic ancestors (Fig. 2c). For this step, we use the calculations of Coop (2013) for generationwise probabilities of genetic ancestry.

### Genetic ancestors and recombination

Coop (2013) used a model of recombination in humans to evaluate the probability that 2 individuals with an ancestor–descendant relationship share at least 1 piece of DNA. In other words, the model gives an approximate probability that a descendant separated by *k* generations from a genealogical ancestor possesses at least 1 genomic fragment from the ancestor. The model takes into account approximations to the recombination process.

In the model of Coop (2013), the number of genomic fragments that a genealogical ancestor passes to a descendant *k* generations forward in time is treated as a random variable $N_{k}$. This random variable is approximated as Poisson-distributed owing to an assumption that recombination breakpoints are Poisson-distributed. The probability $p_{k}$ that a genealogical ancestor is a genetic ancestor to a *k*-generation descendant then equals $1-P[N_{k}=0]=1-e^{-\lambda_{k}}$, where $\lambda_{k}$ is the Poisson mean $E[N_{k}]$.

Considering the autosomal genome, the mean number of genomic pieces that a parent passes to its offspring, $\lambda_{1}$, is 22, the number of autosomes. Each generation, on average every 100 megabases (Mb) a crossover event occurs, adding 1 piece. Because the haploid genome is about 3,300 Mb long, each generation after the first, 33 pieces are added on average. In each generation back in time after the first, those pieces are distributed between 2 parents. Hence, in generation k≥2, the total number of pieces for one of an individual’s 2 genomic copies, maternal or paternal, is 22+33(k−1). Those pieces trace to $2^{k-1}$ genealogical ancestors *k* generations back from the present. Hence, the mean number of fragments contributed by a specific ancestor *k* generations back from the present is $\lambda_{k}=[22+33(k-1)]/2^{k-1}$. The Poisson probability that *at least 1* fragment traces to such an ancestor then equals 1 minus the probability that no fragments trace to the ancestor, or for k≥2,


(1) $$p_{k}=1-e^{-\frac{22+33(k-1)}{2^{k-1}}}.$$

We also define $p_{1}=1$.

**Fig. 3.**
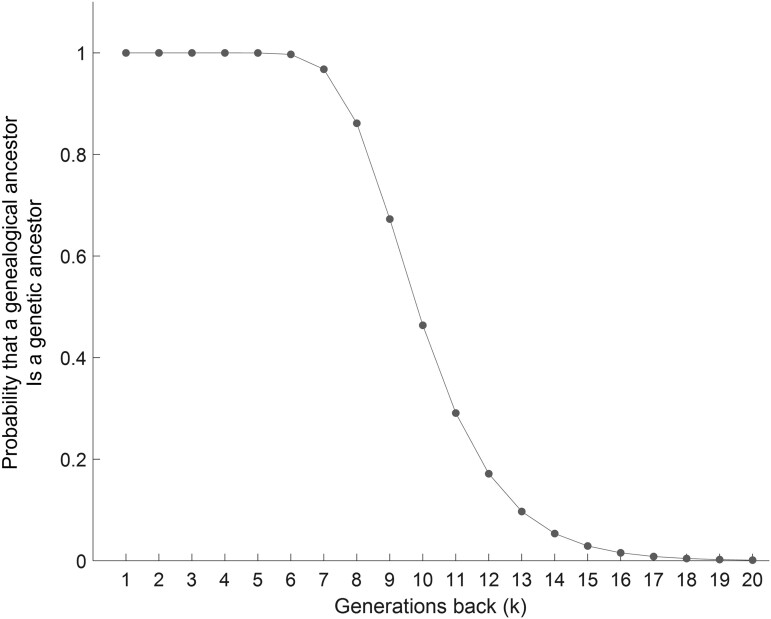
The probability $p_{k}$ that a genealogical ancestor is an (autosomal) genetic ancestor as a function of the number of generations back in time from the present. This plot is based on Eq. 1.

Figure 3 shows $p_{k}$ across the generations, illustrating its decline as *k* increases. With a 25-year generation time, the claim (Donnelly 1983) that an individual living in 1983, say, born in 1960, probably possesses genetic material from a randomly chosen genealogical ancestor born in 1759 corresponds to 8 generations and $p_{8}=0.8615$. The claim that the individual probably does not possess genetic material from a randomly chosen genealogical ancestor born in 1564 corresponds to 16 generations and $p_{16}=0.0157$. Interestingly, the period in which this probability of sharing genetic material with an ancestor decreases from a high to a low number corresponds to the period of interest in the founding of the African-American population, on which our example analysis focuses.

The human-specific Eq. 1 can be written in a more general form suitable for other diploid organisms. Denote the number of pairs of autosomes by *q* and the haploid genome length in megabases by ℓ. Denote by *m* the distance in megabases over which the mean number of crossover events is 1. As in the special case for humans, $p_{1}=1$. The probability that a *k*-generation (k>1) genealogical ancestor is also a genetic ancestor is


(2) $$p_{k}=1-e^{-\frac{q+(\ell /m)(k-1)}{2^{k-1}}}.$$

The computation requires basic parameters of genomes and recombination maps, quantities that are available for diverse organisms (Milo and Phillips 2015; Stapley *et al.* 2017).

## Results for the general model

To count genetic ancestors from source populations in a pedigree of a random admixed individual, we first trace the pedigree back, counting admixed individuals. We then use the count of admixed genealogical ancestors to count genetic ancestors. We also show how this approach can be used to recover the distribution of the number of genealogical ancestors from source populations in each generation, extending beyond calculations from Mooney *et al.* (2023) that focused on the expectation.

### Counting admixed individuals in a pedigree

Continuing to consider a model with *g* generations, we now index generations by *k*, setting k=0 in generation *g*, with *k* increasing backward in time. Let $X_{k}$ be the random number of admixed individuals in the pedigree at step *k*. When k=0, we consider a random admixed individual of interest in generation *g*, and $X_{0}=1$. For 1≤k≤g, we proceed backward in time. At step *k*, or generation g−k, a randomly chosen parent of an admixed individual in the previous step, or generation g−(k−1), has probability $h_{g-k}$ of being an admixed individual. Consequently, because an individual has 2 parents, $X_{k}∼\mathrm{Bin}(2X_{k-1},h_{g-k})$.

The number of admixed individuals in the pedigree is a nonhomogeneous branching process going back in time. It follows from Appendix A that for 0≤k≤g,


(3) $$E[X_{k}]=2^{k}\prod_{i=1}^{k}h_{g-i},$$

(4) $$\begin{array}{rl} \mathrm{Var}[X_{k}] & =\sum_{i=1}^{k}2^{k-1+i}[1-h_{g-(k+1)+i}]\\ & \;\times \left[\left(\prod_{\;j=g-(k+1)+i}^{g-1}h_{j}\right)\left(\prod_{\ell =g-k}^{g-(k+2)+i}h_{\ell }^{2}\right)\right]. \end{array}$$

For the sum of the number of admixed genealogical ancestors across all generations, computing the variance of the sum in Appendix A, we have


(5) $$E\left[\sum_{k=1}^{g}X_{k}\right]=\sum_{k=1}^{g}E[X_{k}],$$

(6) $$\begin{array}{rl} \mathrm{Var}\left[\sum_{k=1}^{g}X_{k}\right] & =\sum_{k=1}^{g}\mathrm{Var}[X_{k}]\\ & \;+\sum_{m=1}^{g-1}\sum_{n=m+1}^{g}\left(2^{n-m+1}\prod_{i=1}^{n-m}h_{g-(m+i)}\mathrm{Var}[X_{m}]\right). \end{array}$$

### Genealogical ancestors

In step *k*, 1≤k≤g, let $U_{k}^{i}$ be the random number of source-1 genealogical ancestors of the generation-*g* admixed individual who are parents of individual *i*, one of the $X_{k-1}$ admixed genealogical individuals in step k−1. Proceeding back in time, after step *k*, $\sum_{\ell =1}^{k}\sum_{i=1}^{X_{\ell -1}}U_{\ell }^{i}$ genealogical ancestors from source 1 have been counted (Fig. 2b).

Random variable $U_{k}^{i}$ takes values 0, 1, and 2, with probabilities as follows:


(7) $$U_{k}^{i}=\left\{ \begin{array}{cc} 0, & h_{g-k}^{2}+2h_{g-k}s_{2,g-k}+s_{2,g-k}^{2},\\ 1, & 2s_{1,g-k}h_{g-k}+2s_{1,g-k}s_{2,g-k},\\ 2, & s_{1,g-k}^{2}. \end{array}\right.$$

In fact, $U_{k}^{i}∼\mathrm{Bin}(2,s_{1,g-k})$, as $1-s_{1,g-k}=h_{g-k}+s_{2,g-k}$. The number of source-2 genealogical ancestors can be counted symmetrically by transposing subscripts 1 and 2 in Eq. 7.

The $\{U_{k}^{i}{\}}_{i=1}^{X_{k-1}}$ are independent and identically distributed. Therefore, using $U_{k}=\sum_{i=1}^{X_{k-1}}U_{k}^{i}$ to sum across all $X_{k-1}$ admixed genealogical ancestors in step k−1, we have for each *k*, 1≤k≤g,


(8) $$U_{k}∼\mathrm{Bin}(2X_{k-1},s_{1,g-k}).$$

Indeed, considering all parents of the admixed individuals in generation g−(k−1), the distribution of the vector of counts of genealogical ancestors in source population 1, the admixed population, and source population 2 can be summarized by a multinomial distribution. If we denote by $U_{k}^{\prime }$ the number of source-2 genealogical ancestors reached in generation g−k, then


(9) $$(U_{k},X_{k},U_{k}^{\prime })∼{\mathrm{Mult}}_{3}[2X_{k-1},(s_{1,g-k},h_{g-k},s_{2,g-k})].$$

By Eq. 3,


(10) $$\begin{array}{rl} E[U_{k}] & =E[E[U_{k}\;|\;X_{k-1}]]=2s_{1,g-k}E[X_{k-1}]\\ & =2^{k}s_{1,g-k}\prod_{i=1}^{k-1}h_{g-i}. \end{array}$$

This equation accords with the summand in Eq. 12 of Mooney *et al.* (2023), noting that generation *i* in that equation is equivalent to generation g−k in Eq. 10. If we consider all $2^{k}$ genealogical ancestors of the generation-*g* admixed individual present in step *k*, 1≤k≤g, then the expected fraction of them who are source-1 individuals who are parents of step-(k−1) admixed individuals is $E[U_{k}]/2^{k}$.

We calculate the variance using the law of total variance together with Eqs. 3 and 4:


(11) $$\begin{array}{rl} \mathrm{Var}[U_{k}] & =E[\mathrm{Var}[U_{k}\;|\;X_{k-1}]]+\mathrm{Var}[E[U_{k}\;|\;X_{k-1}]]\\ & =2s_{1,g-k}(1-s_{1,g-k})E[X_{k-1}]\\ & \;+(2s_{1,g-k}{)}^{2}\mathrm{Var}[X_{k-1}]\\ & =2^{k}s_{1,g-k}(1-s_{1,g-k})\prod_{i=1}^{k-1}h_{g-i}\\ & \;+s_{1,g-k}^{2}\sum_{i=1}^{k-1}2^{k+i}(1-h_{g-k+i})\\ & \;\times \left[\left(\prod_{\;j=g-k+i}^{g-1}h_{j}\right)\left(\prod_{\ell =g-(k-1)}^{g-(k+1)+i}h_{\ell }^{2}\right)\right]. \end{array}$$

We write ${\tilde{s}}_{1,g-k}=s_{1,g-k}/(1-h_{g-k})$ for convenience. Summing genealogical ancestors across generations in Eqs. 10 and 11 and computing the variance in Appendix B, we have


(12) $$E\left[\sum_{k=1}^{g}U_{k}\right]=\sum_{k=1}^{g}E[U_{k}],$$

(13) $$\begin{array}{rl} \mathrm{Var}\left[\sum_{k=1}^{g}U_{k}\right] & =\sum_{k=1}^{g-1}[2{\tilde{s}}_{1,g-(k+1)}-{\tilde{s}}_{1,g-k}{]}^{2}\;\mathrm{Var}[X_{k}]\\ & \;+\left[\sum_{m=1}^{g-2}\sum_{n=m+1}^{g-1}2^{n-m+1}[2{\tilde{s}}_{1,g-(m+1)}-{\tilde{s}}_{1,g-m}\right]\\ & \;\times [2{\tilde{s}}_{1,g-(n+1)}-{\tilde{s}}_{1,g-n}]\\ & \;\times \prod_{i=1}^{n-m}h_{g-(m+i)}\;\mathrm{Var}[X_{m}]]\\ & \;+\sum_{k=0}^{g-1}2s_{1,g-(k+1)}[1-{\tilde{s}}_{1,g-(k+1)}]E[X_{k}]. \end{array}$$

### Genetic ancestors

Next, we count genetic ancestors. Let $Y_{k}^{i}$ be the number of source-1 genetic ancestors of the generation-*g* admixed individual who are parents of individual *i*, one of the admixed genealogical ancestors in step k−1. Proceeding back in time, after step *k*, $\sum_{\ell =1}^{k}\sum_{i=1}^{X_{\ell -1}}Y_{\ell }^{i}$ genetic ancestors from source 1 have been counted. We have for 1≤k≤g probabilities


$$Y_{k}^{i}=\left\{ \begin{array}{cc} 0, & h_{g-k}^{2}+2h_{g-k}s_{2,g-k}+s_{2,g-k}^{2}\\ & \;+s_{1,g-k}^{2}(1-p_{k}{)}^{2}+2s_{1,g-k}h_{g-k}(1-p_{k})\\ & \;+2s_{1,g-k}s_{2,g-k}(1-p_{k}),\\ 1, & 2s_{1,g-k}s_{2,g-k}p_{k}+2s_{1,g-k}h_{g-k}p_{k}\\ & \;+2s_{1,g-k}^{2}p_{k}(1-p_{k}),\\ 2, & s_{1,g-k}^{2}p_{k}^{2}. \end{array}\right.$$

Here, $p_{k}$ is the probability that a genealogical ancestor *k* generations ago is also a genetic ancestor (Eq. 1). The count of genetic ancestors from source 2 is obtained symmetrically.

We can also see that $Y_{k}^{i}∼\mathrm{Bin}(2,s_{1,g-k}p_{k})$, as


$$\begin{array}{rl} P[Y_{k}^{i}=1] & =2s_{1,g-k}p_{k}(1-s_{1,g-k}p_{k}),\\ P[Y_{k}^{i}=2] & =(s_{1,g-k}p_{k}{)}^{2}. \end{array}$$

We write $Y_{k}=\sum_{i=1}^{X_{k-1}}Y_{k}^{i}$ for the number of genetic ancestors tabulated in step *k*. By analogy with the tabulation of genealogical ancestors, we conclude by Eqs. 3 and 4 that for 1≤k≤g,


(14) $$Y_{k}∼\mathrm{Bin}(2X_{k-1},s_{1,g-k}p_{k}),$$

(15) $$E[Y_{k}]=2^{k}s_{1,g-k}p_{k}\prod_{i=1}^{k-1}h_{g-i},$$

(16) $$\begin{array}{rl} \mathrm{Var}[Y_{k}] & =2^{k}s_{1,g-k}p_{k}(1-s_{1,g-k}p_{k})\prod_{i=1}^{k-1}h_{g-i}\\ & \;+(s_{1,g-k}p_{k}{)}^{2}\sum_{i=1}^{k-1}2^{k+i}(1-h_{g-k+i})\\ & \;\times \left[\left(\prod_{\;j=g-k+i}^{g-1}h_{j}\right)\left(\prod_{\ell =g-(k-1)}^{g-(k+1)+i}h_{\ell }^{2}\right)\right]. \end{array}$$

For the sum of the number of genetic ancestors across all generations, computing the variance in Appendix B, we have


(17) $$\begin{array}{rl} E\left[\sum_{k=1}^{g}Y_{k}\right] & =\sum_{k=1}^{g}E[Y_{k}], \end{array}$$

(18) $$\begin{array}{rl} \mathrm{Var}\left[\sum_{k=1}^{g}Y_{k}\right] & =\sum_{k=1}^{g-1}[2{\tilde{s}}_{1,g-(k+1)}p_{k+1}-{\tilde{s}}_{1,g-k}p_{k}{]}^{2}\\ & \;\times \mathrm{Var}[X_{m}]+[\sum_{m=1}^{g-2}\sum_{n=m+1}^{g-1}2^{n-m+1}\\ & \;\times [2{\tilde{s}}_{1,g-(m+1)}p_{m+1}-{\tilde{s}}_{1,g-m}p_{m}]\\ & \;\times [2{\tilde{s}}_{1,g-(n+1)}p_{n+1}-{\tilde{s}}_{1,g-n}p_{n}]\\ & \;\times \prod_{i=1}^{n-m}h_{g-(m+i)}\;\mathrm{Var}[X_{m}]]\\ & \;+\sum_{k=0}^{g-1}2s_{1,g-(k+1)}p_{k+1}\\ & \;\times [1-{\tilde{s}}_{1,g-(k+1)}p_{k+1}]E[X_{k}]. \end{array}$$

Among all $2^{k}$ genealogical ancestors of the generation-*g* admixed individual who are present in step *k*, 1≤k≤g, the expected fraction of them who are source-1 individuals who are parents of step-(k−1) admixed individuals and are genetic ancestors is $E[Y_{k}]/2^{k}$.

In the same way that we count genetic ancestors among the genealogical ancestors from the source populations, we can count the number of admixed genealogical ancestors who are also genetic ancestors. Denoting the random number of admixed genetic ancestors in step *k* by $X_{k}^{*}$, this random variable is binomially distributed for 1≤k≤g, so that


(19) $$X_{k}^{*}∼\mathrm{Bin}(2X_{k-1},h_{g-k}p_{k}),$$

(20) $$E[X_{k}^{*}]=2^{k}h_{g-k}p_{k}\prod_{i=1}^{k-1}h_{g-i},$$

(21) $$\begin{array}{rl} \mathrm{Var}[X_{k}^{*}] & =2^{k}h_{g-k}p_{k}(1-h_{g-k}p_{k})\prod_{i=1}^{k-1}h_{g-i}\\ & \;+(h_{g-k}p_{k}{)}^{2}\sum_{i=1}^{k-1}2^{k+i}(1-h_{g-k+i})\\ & \;\times \left[\left(\prod_{\;j=g-k+i}^{g-1}h_{j}\right)\left(\prod_{\ell =g-(k-1)}^{g-(k+1)+i}h_{\ell }^{2}\right)\right]. \end{array}$$

The expected fraction of the $2^{k}$ genealogical ancestors of the generation-*g* admixed individual who are themselves admixed individuals and who are also genetic ancestors is $E[X_{k}^{*}]/2^{k}$.

Considering all parents of the admixed individuals in generation g−(k−1), the distribution of the vector of counts of genetic ancestors in source population 1, the admixed population, and source population 2 follows a multinomial distribution. If we denote by $Y_{k}^{\prime }$ the number of source-2 genealogical ancestors reached in generation g−k, then


(22) $$(Y_{k},X_{k}^{*},Y_{k}^{\prime })∼{\mathrm{Mult}}_{3}[2X_{k-1},(s_{1,g-k}p_{k},h_{g-k}p_{k},s_{2,g-k}p_{k})].$$

For the sum of the number of genetic ancestors across generations, we have


(23) $$E\left[\sum_{k=1}^{g}X_{k}^{*}\right]=\sum_{k=1}^{g}E[X_{k}^{*}],$$

(24) $$\begin{array}{rl} \mathrm{Var}\left[\sum_{k=1}^{g}X_{k}^{*}\right] & =\sum_{k=1}^{g}\mathrm{Var}[X_{k}^{*}]+\sum_{m=1}^{g-1}\sum_{n=m+1}^{g}2^{n-m+1}\\ & \;\times \prod_{i=1}^{n-m}h_{g-(m+i)}p_{m+i}\;\mathrm{Var}[X_{m}^{*}]. \end{array}$$

## A single admixture event

We now consider 2 specific cases of the admixture model, where after the initial generation of admixture, the contributions from the 2 sources and from the admixed population are constant across generations. First, we study the case in which the constants are 0. We examine the situation in which no subsequent admixture occurs after the admixed population is founded: in other words, $s_{1,0},s_{2,0}>0$ and for all *n*, 1≤n≤g−1, $s_{1,n}=s_{2,n}=0$ and $h_{n}=1$.

For each k=1,2,…,g−1, the random number of admixed individuals in the pedigree of a randomly chosen admixed individual follows $X_{k}∼\mathrm{Bin}(2X_{k-1},1)$. Recalling that $X_{0}=1$ for the single admixed individual in generation *g*, we have $X_{k}=2^{k}$ for all k=0,1,2,…,g−1: all $2^{k}$ ancestors of an individual *k* generations back from the present are admixed.

To consider genealogical ancestors from the source populations, we separate between 2 cases, 1≤k≤g−1 and k=g. For 1≤k≤g−1, $U_{k}∼\mathrm{Bin}(2^{k},0)$ and no individuals from sources 1 and 2 are reached. Consequently, $U_{k}=0$ for all *k* with 1≤k≤g−1.

Next, we proceed one generation back from the case of k=g−1. If k=g, then by Eq. 8, $U_{g}∼\mathrm{Bin}(2\cdot 2^{g-1},s_{1,0})$. Therefore, $E[U_{g}]=2^{g}s_{1,0}$ and $\mathrm{Var}[U_{g}]=2^{g}s_{1,0}(1-s_{1,0})$.

For genetic ancestors, we again separate 1≤k≤g−1 from k=g. For 1≤k≤g−1, $Y_{k}∼\mathrm{Bin}(2^{k},0)$, and the count of genetic ancestors is $Y_{k}=0$ for all *k* with 1≤k≤g−1, as is seen with genealogical ancestors. For k=g, by Eq. 14, $Y_{g}∼\mathrm{Bin}(2^{g},s_{1,0}p_{g})$. Therefore, $E[Y_{g}]=2^{g}s_{1,0}p_{g}$ and $\mathrm{Var}[Y_{g}]=2^{g}s_{1,0}p_{g}(1-s_{1,0}p_{g})$. The numbers of genetic ancestors from the source populations, like the corresponding numbers of genealogical ancestors, are determined by parameters of the initial admixture, as tabulated by n=0 looking forward in time, or by k=g looking backward.

## Constant positive admixture

We now examine the situation in which $s_{1,0},s_{2,0}>0$, after which the contributions from the sources are constant and positive. We denote $s_{1,n}=s_{1}$ and $s_{2,n}=s_{2}$ for all *n*, 1≤n≤g−1, with $s_{1},s_{2}>0$. Then $h_{n}=1-s_{1,n}-s_{2,n}$ is also constant for all *n*, 1≤n≤g−1; we denote this constant by $h_{n}=h$.

### Mathematical results

The number of admixed genealogical ancestors $X_{k}$ follows a homogeneous branching process. For k=0, $E[X_{k}]=1$. By Eq. 3, for k=1,2,…,g−1,


(25) $$E[X_{k}]=(2h{)}^{k}.$$

For k=g, $E[X_{k}]=0$.

For the variance of the number of admixed genealogical ancestors, by Eq. 4, $\mathrm{Var}[X_{0}]=0$ and for 1≤k≤g−1,


(26) $$\begin{array}{rl} \mathrm{Var}[X_{k}] & =\sum_{i=1}^{k}2^{k-1+i}(1-h)\\ & \;\times \left[\left(\prod_{\;j=g-(k+1)+i}^{g-1}h\right)\left(\prod_{\ell =g-k}^{g-(k+2)+i}h^{2}\right)\right]\\ & =\sum_{i=1}^{k}(1-h)2^{k-1+i}h^{k-1+i}\\ & =\left\{ \begin{array}{cc} \frac{1-h}{1-2h}(2h{)}^{k}[1-(2h{)}^{k}],\; & h\neq \frac{1}{2},\;\\ \frac{k}{2},\; & h=\frac{1}{2}.\; \end{array}\right. \end{array}$$

For k=g, $\mathrm{Var}[X_{k}]=0$.

To count genealogical and genetic ancestors, we again separate 1≤k≤g−1 from k=g. When k=g, by Eq. 8, $U_{g}∼\mathrm{Bin}(2X_{g-1},s_{1,0})$. Hence, by Eqs. 10 and 25, for genealogical ancestors, we have


(27) $$E[U_{g}]=2^{g}h^{g-1}s_{1,0}.$$

For the variance, starting from Eq. 11 and applying Eqs. 25 and 26, we have


(28) $$\begin{array}{rl} \mathrm{Var}[U_{g}] & =2s_{1,0}(1-s_{1,0})E[X_{g-1}]+(2s_{1,0}{)}^{2}\mathrm{Var}[X_{g-1}]\\ & =\left\{ \begin{array}{cc} 2s_{1,0}(1-s_{1,0})2^{g-1}h^{g-1}\\ \;+(2s_{1,0}{)}^{2}\left(\frac{1-h}{1-2h}\right)(2h{)}^{g-1}[1-(2h{)}^{g-1}], & h\neq \frac{1}{2},\;\\ 2s_{1,0}(1-s_{1,0})2^{g-1}{\left(\frac{1}{2}\right)}^{g-1}+(2s_{1,0}{)}^{2}\left(\frac{g-1}{2}\right), & h=\frac{1}{2}.\; \end{array}\right.\\ & =\left\{ \begin{array}{cc} 2s_{1,0}(2h{)}^{g-1}\\ \;\times \{1-s_{1,0}+2s_{1,0}\left(\frac{1-h}{1-2h}\right)[1-(2h{)}^{g-1}]\}, & h\neq \frac{1}{2},\;\\ 2s_{1,0}[1+s_{1,0}(g-2)], & h=\frac{1}{2}.\; \end{array}\right. \end{array}$$

For 1≤k≤g−1, by Eq. 8, $U_{k}∼\mathrm{Bin}(2X_{k-1},s_{1})$. By Eqs. 10 and 25,


(29) $$E[U_{k}]=2^{k}h^{k-1}s_{1}.$$

We then obtain, by Eqs. 11, 25, and 26,


(30) $$\mathrm{Var}[U_{k}]=\left\{ \begin{array}{cc} 2s_{1}(2h{)}^{k-1}\\ \;\times \{1-s_{1}+2s_{1}\left(\frac{1-h}{1-2h}\right)[1-(2h{)}^{k-1}]\}, & h\neq \frac{1}{2},\;\\ 2s_{1}[1+s_{1}(k-2)], & h=\frac{1}{2}.\; \end{array}\right.$$

For genetic ancestors, when k=g, similarly to the calculations for genealogical ancestors, we use Eq. 14 to obtain $Y_{g}∼\mathrm{Bin}(2X_{g-1},s_{1,0}p_{g})$. By Eqs. 15 and 25,


(31) $$E[Y_{g}]=2^{g}h^{g-1}s_{1,0}p_{g}.$$

Following the reasoning underlying Eq. 16, with Eqs. 25 and 26,


(32) $$\begin{array}{rl} \mathrm{Var}[Y_{g}] & =2s_{1,0}p_{g}(1-s_{1,0}p_{g})E[X_{g-1}]+(2s_{1,0}p_{g}{)}^{2}\mathrm{Var}[X_{g-1}]\\ & =\left\{ \begin{array}{cc} 2s_{1,0}p_{g}(2h{)}^{g-1}\\ \;\times \{1-s_{1,0}p_{g}+2s_{1,0}p_{g}\left(\frac{1-h}{1-2h}\right)[1-(2h{)}^{g-1}]\}, & h\neq \frac{1}{2},\;\\ 2s_{1,0}p_{g}[1+s_{1,0}p_{g}(g-2)], & h=\frac{1}{2}.\; \end{array}\right. \end{array}$$

For 1≤k≤g−1, by Eq. 14, $Y_{k}∼\mathrm{Bin}(2X_{k-1},s_{1}p_{k})$. Hence, by Eqs. 15 and 25,


(33) $$E[Y_{k}]=2^{k}h^{k-1}s_{1}p_{k}.$$

By Eqs. 16, 25, and 26,


(34) $$\mathrm{Var}[Y_{k}]=\left\{ \begin{array}{cc} 2s_{1}p_{k}(2h{)}^{k-1}\\ \;\times \{1-s_{1}p_{k}+2s_{1}p_{k}\left(\frac{1-h}{1-2h}\right)[1-(2h{)}^{k-1}]\}, & h\neq \frac{1}{2},\;\\ 2s_{1}p_{k}[1+s_{1}p_{k}(k-2)], & h=\frac{1}{2}.\; \end{array}\right.$$

### Analysis of temporal trends

In the case of constant positive admixture, we analyze the way in which genealogical and genetic ancestors accumulate across the generations of the admixture process. Comparing generation *k*, 2≤k≤g−1, to the generation k−1 of its offspring, Eq. 29 gives


$$\frac{E[U_{k}]}{E[U_{k-1}]}=2h.$$

**Fig. 4.**
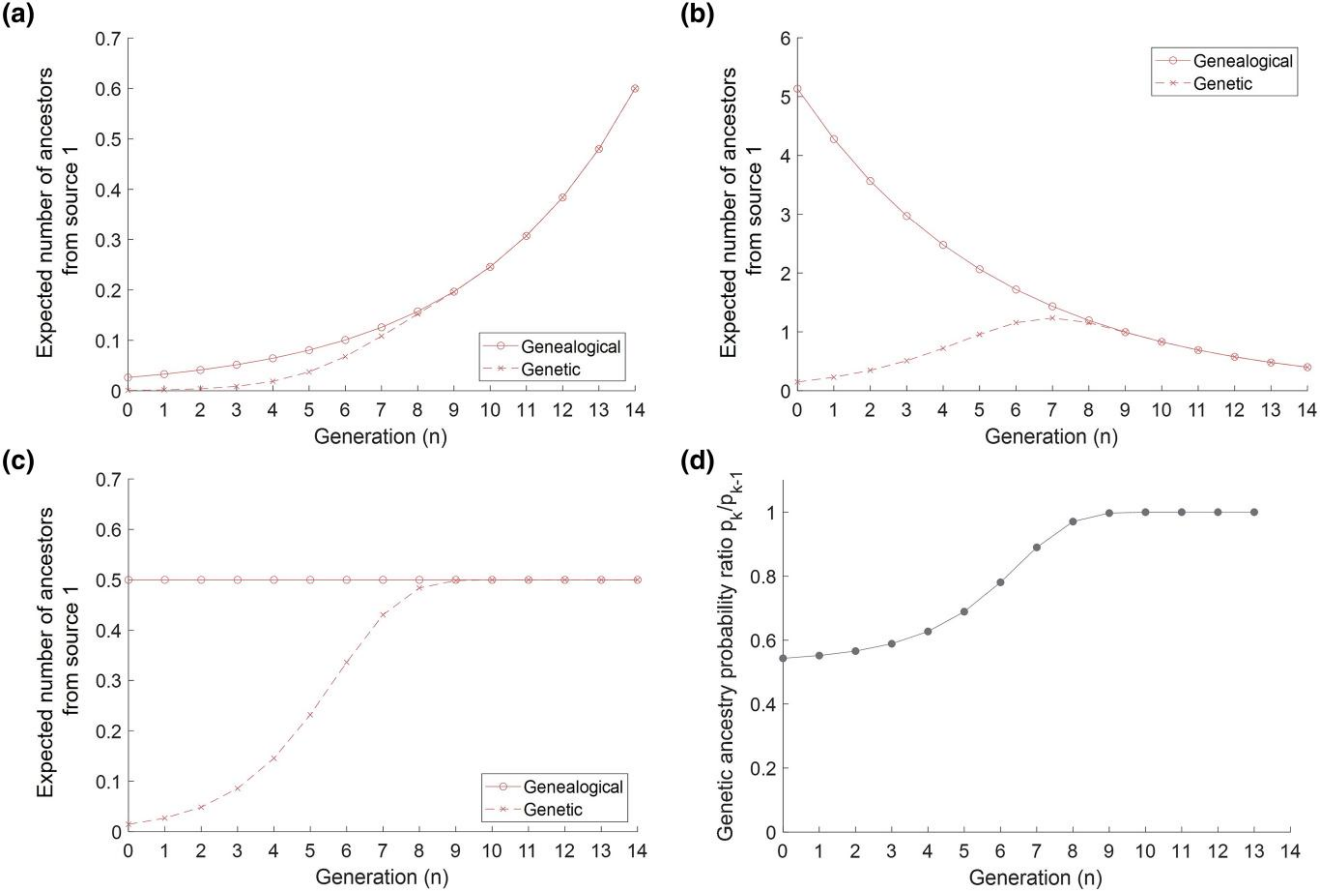
Genealogical and genetic ancestors in a model of constant admixture with g=15, evaluated forward in time from generation n=0 to generation n=g−1=14. The forward-time generation *n* corresponds to the backward-time generation k=g−n. a–c) Expected number of source-1 genealogical ancestors (Eqs. 27, 29) and genetic ancestors (Eqs. 31, 33). The 3 panels use $s_{1,0}=s_{2,0}=0.5$ and $s_{1}=s_{2}$ with different values of *h*. a) h=0.4. b) h=0.6. c) h=0.5. d) The ratio of the conditional probabilities of genetic ancestry given genealogical ancestry for generations *k* and k−1, $\;p_{k}/\;p_{k-1}$ (Eq. 1), where k=0 in generation g=15 and n=g−k. Note that this plot stops at n=13 and k=2 with the value of $p_{2}/p_{1}$.

If $h<\frac{1}{2}$, then 2h<1 and $E[U_{k}]$ decreases with increasing *k* and hence decreasing n=g−k (Fig. 4a). The number of admixed ancestors is small, so that the source populations are likely to be reached in a small number of generations back from the present; hence, the numbers of genealogical ancestors from the source populations are also small. The contribution from the admixed population is low enough and the contributions from the source populations are high enough that the number of genealogical ancestors from the source populations is greatest in the most recent generations.

If, on the other hand, $h>\frac{1}{2}$, then 2h>1 and $E[U_{k}]$ increases with increasing *k* and decreasing n=g−k (Fig. 4b). The number of admixed genealogical ancestors is larger than with $h<\frac{1}{2}$, so that the number of genealogical ancestors from the source populations is also larger. With a high contribution from the admixed population to itself, the number of genealogical ancestors from the source populations is greatest farther back in time. A transition occurs at $h=\frac{1}{2}$, where 2h=1 and $E[U_{k}]$ is constant in time, equaling $2s_{1}$ by Eq. 29 (Fig. 4c).

For genetic ancestors, for 2≤k≤g−1, Eq. 33 gives


$$\frac{E[Y_{k}]}{E[Y_{k-1}]}=2h\frac{\;p_{k}}{p_{k-1}}.$$

Although the admixture process is constant in time after the founding of the admixed population, the dependence of $p_{k}$ on *k* (Eq. 1) affects the time at which genetic ancestors from the sources accumulate.

We now examine $\;p_{k}/\;p_{k-1}$. Denote the event “a *k*-generation genealogical ancestor is a *k*-generation genetic ancestor” by $A_{k}$, k≥1. Irrespective of the form chosen for $P[A_{k}]$, we argue that


(35) $$\frac{1}{2}\leq \frac{P[A_{k}]}{P[A_{k-1}]}=\frac{\;p_{k}}{p_{k-1}}\leq 1.$$

For the right-hand side of Eq. 35, a necessary condition for a *k*-generation genealogical ancestor of a descendant to be a *k*-generation genetic ancestor is that it is a (k−1)-generation genetic ancestor of the parent of the descendant. In other words, $A_{k}\subseteq A_{k-1}$ and $P[A_{k}]\leq P[A_{k-1}]$.

For the left-hand side of Eq. 35, because $A_{k}\subseteq A_{k-1}$,


$$\frac{P[A_{k}]}{P[A_{k-1}]}=\frac{P[A_{k}\cap A_{k-1}]}{A_{k-1}}=P[A_{k}\;|\;A_{k-1}].$$

$A_{k}\;|\;A_{k-1}$ is the event that conditional on a *k*-generation ancestor transmitting at least 1 genomic segment to the parent of a descendant, the *k*-generation ancestor transmits at least 1 segment to the descendant itself. The probability that a parent transmits a certain segment to an offspring is $\frac{1}{2}$, and therefore $\frac{1}{2}\leq P[A_{k}\;|\;A_{k-1}]$.

For the functional form of $P[A_{k}]$ used by Coop (2013), Eq. 1, a proof that $\frac{1}{2}<p_{k}<1$ for all k≥2 appears in Appendix C. An example of $p_{k}/p_{k-1}$ appears in Fig. 4d, illustrating a decrease in $p_{k}/p_{k-1}$ with increasing *k* and decreasing n=g−k.

## Application to African-Americans

### Model and methods

We apply our model to count genetic ancestors for a random individual in the African-American population in the United States. In Mooney *et al.* (2023), relying on demographic data on the history of the population, we considered a model with g=14 generations, ending in 1960–1965. Using information on current patterns of genetic admixture, we inferred admixture parameters $\left(s_{1,n},h_{n},s_{2,n}\right)$, with source 1 representing Africans and source 2 representing Europeans. The model divided the demographic history of the population into 3 epochs: 1619–1808, during which the population was founded, with importation of enslaved African captives and admixture with Europeans; 1808–1865, during which enslavement and admixture continued but importation of enslaved persons was illegal; and 1865–1965, after the end of legal enslavement. The 1965 endpoint for the model was chosen to accord with the approximate timing of the birth of individuals in whom genetic ancestry has been measured, and to precede subsequent major demographic changes.

The model considered 25-year generations, initializing the population solely with Africans ($s_{1,0}=1,s_{2,0}=0$). The first epoch had 7 generations (1635–1640, 1660–1665, 1685–1690, 1710–1715, 1735–1740, 1760–1765, 1785–1790; n=1–7), the second epoch had 3 (1810–1815, 1835–1840, 1860–1865; n=8–10) and the third had 4 (1885–1890, 1910–1915, 1935–1940, 1960–1965; n=11–14). In the first epoch, $s_{2,n}$ was kept constant, and the values of $s_{1,n}$ and $h_{n}$ were specified by estimating the value of $s_{1,n}/(s_{1,n}+h_{n})$ using demographic data (Hacker 2020) about newly transported enslaved individuals from Africa and births in the African-American population. In both the second and third epochs, $s_{1,n}$, $h_{n}$, and $s_{2,n}$ were maintained as constants for all generations in the epoch.

**Fig. 5.**
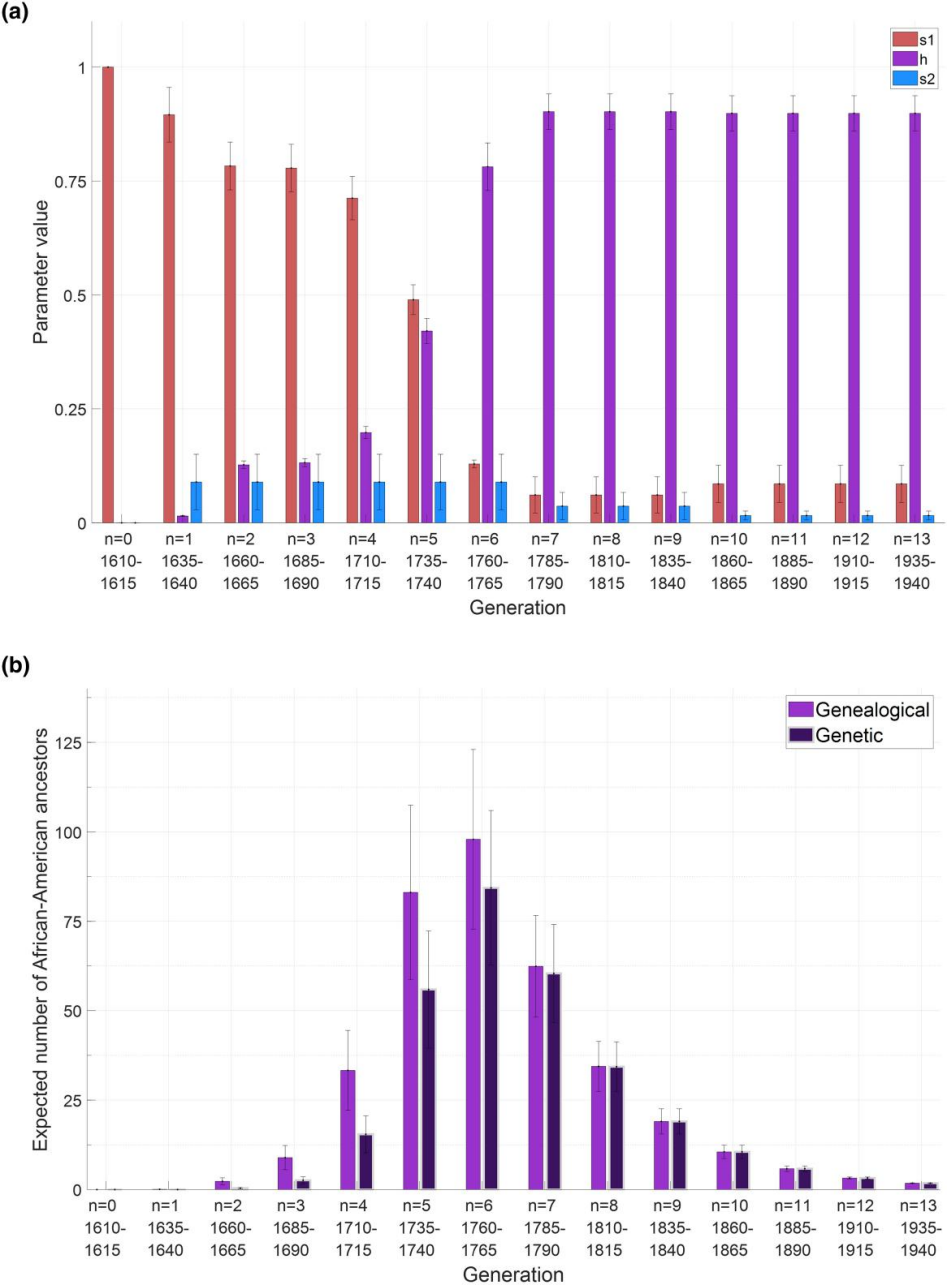
Generation-specific genealogical and genetic ancestry features for African-Americans. a) Generationwise mean admixture contributions $s_{1}$ (African), *h* (African-American), and $s_{2}$ (European) across accepted parameter sets. Error bars show standard deviations. b) Means across accepted parameter sets of the expected numbers of African-American genealogical and genetic ancestors possessed by a random individual, as calculated by Eqs. 3 and 20. Error bars show the standard deviations of these expected numbers across accepted parameter sets. The values plotted in a) are obtained by summarizing the distributions underlying Fig. 4 of Mooney *et al.* (2023). The values in b) are given in Table 1.

Mooney *et al.* (2023) identified sets of parameter values that recovered features of genetic ancestry measured in African-Americans: an expected African genetic ancestry in [0.75,0.85] with standard deviation in [0.08,0.15]. A summary of generationwise mean parameter values across all accepted parameter sets appears in Fig. 5a. The figure reports mean values of $s_{1}$, *h*, and $s_{2}$, summarizing distributions that appear in Fig. 4 of Mooney *et al.* (2023). It shows the high African contribution to the African-American population in the earliest generations ($s_{1}$), with an increasing contribution of the African-American population to itself (*h*), and with European contributions occurring across the generations ($s_{2}$). For each set of accepted parameters, Mooney *et al.* (2023) calculated the generationwise expected numbers of African and European genealogical ancestors associated with the set.

**Table 1.** Generation-specific expectations of the numbers of African-American genealogical and genetic ancestors across accepted parameter sets.

		Number of African-American ancestors					
		Genealogical			Genetic		
		Mean	Standard	Mean of	Mean	Standard	Mean of
Generation	Birth	of	deviation of	standard	of	deviation of	standard
(*n*)	year	expectation	expectation	deviation	expectation	expectation	deviation
0	1610–1615	—	—	—	—	—	—
1	1635–1640	0.07	0.03	0.27	0.01	0.00	0.08
2	1660–1665	2.32	0.98	1.83	0.40	0.17	0.66
3	1685–1690	8.93	3.38	4.41	2.60	0.98	1.88
4	1710–1715	33.30	11.15	12.71	15.44	5.17	6.60
5	1735–1740	83.09	24.37	28.93	55.91	16.39	20.05
6	1760–1765	97.93	25.08	33.17	84.36	21.61	28.99
7	1785–1790	62.39	14.22	21.05	60.39	13.76	20.57
8	1810–1815	34.43	6.97	11.51	34.33	6.95	11.52
9	1835–1840	19.04	3.52	6.28	19.04	3.52	6.28
10	1860–1865	10.55	1.87	3.40	10.55	1.87	3.40
11	1885–1890	5.84	0.77	1.83	5.84	0.77	1.83
12	1910–1915	3.24	0.28	0.94	3.24	0.28	0.94
13	1935–1940	1.80	0.08	0.42	1.80	0.08	0.42
Total	—	362.93	90.16	119.94	293.89	69.66	99.54

Suppose $\theta_{i}$ denotes an accepted parameter set and $\theta =\{\theta_{i}{\}}_{i=1}^{\left|\theta \right|}$ denotes the collection of all accepted parameter sets. For each generation n=g−k with g=14 (k=1,2,…,g), the mean of the expectation of the genealogical ancestors is ${\mathrm{Mean}}_{\theta }\{E[X_{k}(\theta_{i})]\}$ (Eq. 3; Eq. 20 for genetic ancestors); the standard deviation of the expectation is $\sigma_{\theta }\{E[X_{k}(\theta_{i})]\}$; the mean of the standard deviation is ${\mathrm{Mean}}_{\theta }\{\sqrt{\mathrm{Var}[X_{k}(\theta_{i})]}\}$ (Eq. 4; Eq. 21 for the genetic ancestors). For the total, the mean of the expectation of the genealogical ancestors is ${\mathrm{Mean}}_{\theta }\{E[\sum_{k=1}^{g}X_{k}(\theta_{i})]\}$ (Eq. 5; Eq. 23 for the genetic ancestors); the standard deviation of the expectation is $\sigma_{\theta }\{E[\sum_{k=1}^{g}X_{k}(\theta_{i})]\}$; the mean of the standard deviation is ${\mathrm{Mean}}_{\theta }\{\sqrt{\mathrm{Var}[\sum_{k=1}^{g}X_{k}(\theta_{i})]}\}$ (Eq. 6; Eq. 24 for genetic ancestors). The table shows the generationwise values plotted in Fig. 5b for the mean and standard deviation of the expectation.

Here, using these parameter sets, we calculate the generationwise expected numbers of African-American genealogical ancestors and the expected numbers of African, European, and African-American *genetic* ancestors (Eq. 15), in a pedigree of a person drawn randomly from the African-American population born between 1960 and 1965. We also show the distribution across parameter sets, in each generation, of the expected numbers of genealogical ancestors from each population.

### Genealogical ancestors

For each accepted parameter set, using Eq. 3, we evaluated the generationwise expected number of African-American ancestors that appear in a random genealogy, represented by $E[X_{k}]$. The mean across accepted parameter sets is shown in Fig. 5b and Table 1. Forward in time, the mean number of African-American genealogical ancestors is initially small, increasing to a peak in generation 6 (1760–1765) with a value of 98. It decreases toward the end of the admixture process.

At each generation *n*, genealogical ancestry is split across 5 groups: Africans reached in generation *n*, African-Americans present in generation *n*, Europeans reached in generation *n*, Africans who are ancestors to Africans reached subsequent to generation *n*, and Europeans who are ancestors to Europeans reached subsequent to generation *n*. The first and third of these categories were studied by Mooney *et al.* (2023). The fourth and fifth are individuals who are genealogical ancestors of individuals who contributed directly to the African-American population, but who are not themselves parents of African-Americans; the expected number of Africans who are ancestors to African genealogical ancestors reached only subsequent to generation *n* is obtained from Eq. 10 by $\sum_{i=n+1}^{13}2^{i-n}E[U_{14-i}]$. A similar computation can be performed for Europeans.

**Fig. 6.**
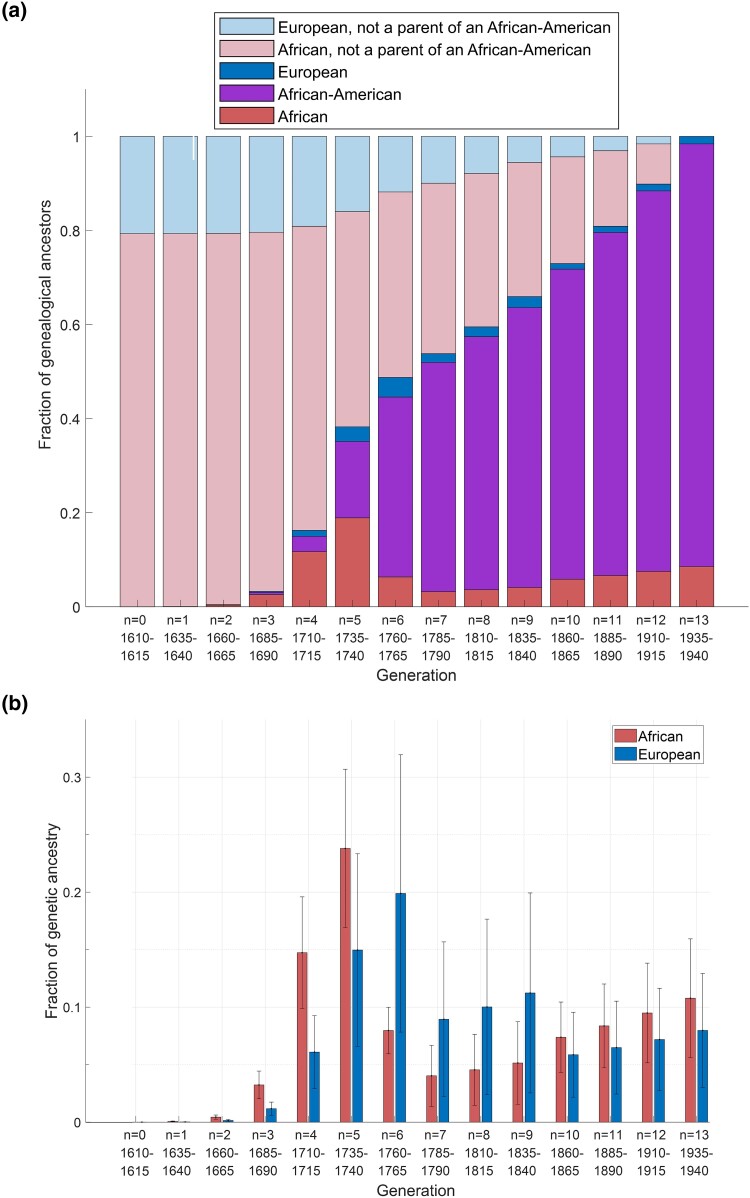
Generation-specific genealogical and genetic ancestry fractions for African-Americans. a) Generationwise genealogical ancestry for a random African-American individual, partitioned across 5 categories and averaged across accepted parameter sets. The fraction of genealogical ancestors who are Africans in generation *n* who contribute directly to the African-American population is obtained from Eq. 10 as $E[U_{14-n}]/2^{14-n}$; the fraction of genealogical ancestors who are African but who only contribute to the African-American population through their subsequent African descendants is $(\sum_{i=n+1}^{13}2^{i-n}E[U_{14-i}])/2^{14-n}$. Similar calculations are performed for Europeans. The fraction of genealogical ancestors who are African-American is $E[X_{14-n}]/2^{14-n}$, calculated using Eq. 3. The values plotted appear in Table 2. b) Generationwise expected African genetic ancestry contributed to a descendant as a fraction of the total expected African genetic ancestry in the descendant, and expected European genetic ancestry contributed to the descendant as a fraction of the total expected European genetic ancestry in the descendant. The values are obtained from Eq. 36, with n=14−k. Error bars represent standard deviations of the values from Eq. 36 across accepted parameter sets.

Figure 6a plots the fractions among all genealogical ancestors assigned to the 5 categories, and the values plotted appear in Table 2. In the earliest generations, all genealogical ancestors are Africans and Europeans who do not directly contribute to the African-American population. As the admixture continues, African and European genealogical ancestors who directly contribute are reached, and eventually, African-Americans represent most of the genealogical ancestors. In generation 0 (1610–1615), ∼79% of genealogical ancestors are African and ∼21% are European, reflecting the fractions of an African-American genome that trace to African genetic ancestry and to European genetic ancestry.

**Table 2.** Generation-specific expectations of the fractions of genealogical ancestry assigned to 5 categories, across accepted parameter sets.

		Fraction of genealogical ancestors				
Generation	Birth	African	African-	European	African,	European,
(*n*)	year		American		not counted	not counted
0	1610–1615	0.0000	—	—	0.7934	0.2066
1	1635–1640	0.0005	0.0000	0.0000	0.7929	0.2066
2	1660–1665	0.0035	0.0006	0.0003	0.7894	0.2062
3	1685–1690	0.0257	0.0044	0.0024	0.7637	0.2038
4	1710–1715	0.1171	0.0325	0.0127	0.6466	0.1911
5	1735–1740	0.1890	0.1623	0.0313	0.4575	0.1599
6	1760–1765	0.0632	0.3825	0.0417	0.3944	0.1182
7	1785–1790	0.0320	0.4874	0.0186	0.3624	0.0996
8	1810–1815	0.0362	0.5380	0.0208	0.3262	0.0788
9	1835–1840	0.0409	0.5950	0.0234	0.2853	0.0554
10	1860–1865	0.0584	0.6593	0.0118	0.2269	0.0436
11	1885–1890	0.0663	0.7296	0.0130	0.1606	0.0305
12	1910–1915	0.0752	0.8088	0.0145	0.0854	0.0161
13	1935–1940	0.0854	0.8985	0.0161	—	—

The table shows the values plotted in Fig. 6a.

### Genetic ancestors

Considering the accepted parameter sets from Mooney *et al.* (2023), we used Eq. 15 to calculate generationwise expected numbers of African and European *genetic* ancestors. These values enable evaluation of expected fractions of the total African and European ancestry that have contributed to a descendant genome by each generation of genetic ancestors. For example, the fraction of the genome that traces to a specific African genetic ancestor from *k* generations before the descendant is, on average, $1/W_{k}$, where $W_{k}$ is the number of genetic ancestors in that generation. $W_{k}$ has expectation $2^{k}p_{k}$, the product of the number of genealogical ancestors *k* generations ago and the probability that a genealogical ancestor is a genetic ancestor. Therefore, the expected contribution to the African genetic ancestry fraction from all African genetic ancestors *k* generations before the present can be approximated by $E[Y_{k}]/(2^{k}p_{k})$, the ratio of the expected number of African genetic ancestors *k* generations prior to the descendant and the expected total number of genetic ancestors in that generation. By Eqs. 10 and 15, $E[Y_{k}]/(2^{k}p_{k})=E[U_{k}]/2^{k}$.

Figure 6b shows the expected African and European genetic ancestry contributed by the genetic ancestors from each generation as fractions of the total African and European genetic ancestry, or


(36) $$\frac{E[Y_{k}]/(2^{k}p_{k})}{\sum_{\ell =1}^{14}E[Y_{\ell }]/(2^{\ell }p_{\ell })}.$$

The figure converts between the backward-time perspective indexed by *k* and the forward-time n=g−k. Because a genetic ancestor from the more recent generations (large *n*) contributes more genetic ancestry on average than a genetic ancestor in previous generations (small *n*), we observe nonnegligible contributions from these later generations. However, ∼40% of the African genetic ancestry traces to generations 4 and 5, and ∼35% of the European genetic ancestry traces to generations 5 and 6, with an additional ∼30% of European genetic ancestry tracing to generations 7, 8, and 9.

**Fig. 7.**
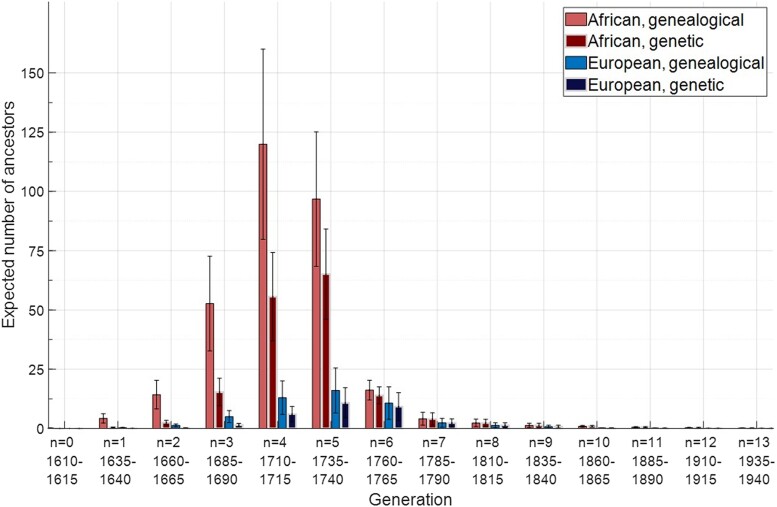
Generation-specific expectations of the numbers of African and European genealogical and genetic ancestors. The expected number of African genealogical ancestors is calculated according to Eq. 10 (standard deviation, Eq. 11). The expected number of African genetic ancestors is calculated according to Eq. 15 (standard deviation, Eq. 16). Similar calculations are performed for Europeans. The plot shows means of the expectation and standard deviation across expected parameter sets. The values plotted appear in Table 4.

The generationwise mean values across parameter sets of the expected numbers of genetic ancestors appear in Fig. 7, alongside expected numbers of genealogical ancestors for comparison. Replotting values from Fig. 7 of Mooney *et al.* (2023), the numbers of genealogical ancestors are greater for Africans than for Europeans, and the expected total numbers of genealogical ancestors, summing across generations, are 314 Africans and 51 Europeans (Tables 3 and 4). Looking forward in time from the founding of the population, the numbers of genealogical ancestors increase to peak values and then decrease. The numbers of genetic ancestors also reach peaks and decrease toward the present. The expected total numbers of genetic ancestors are 162 Africans and 32 Europeans.

**Table 3.** Summary statistics for the expected numbers of African, European, and African-American genealogical and genetic ancestors for a random individual from the African-American population across the accepted parameter sets.

Quantity	Mean	Standard deviation	Minimum	First quartile	Median	Third quartile	Maximum
African genealogical ancestors	314	99	124	240	299	376	680
African genetic ancestors	162	47	72	127	155	192	332
European genealogical ancestors	51	24	4	32	51	69	125
European genetic ancestors	32	14	4	21	32	43	77
African-American genealogical ancestors	363	90	202	294	345	418	709
African-American genetic ancestors	294	70	172	240	280	336	566

The estimates consider random individuals in the 1960–1965 birth cohort, assumed to be generation g=14 in a 3-epoch model. The standard deviations are standard deviations of the means across accepted parameter sets; means and standard deviations are rounded from Tables 1 and 4. The values for African and European genealogical ancestors appear in Table 3 in Mooney *et al.* (2023).

**Table 4.** Generation-specific expectations of the numbers of African and European genealogical and genetic ancestors across accepted parameter sets.

		Number of African ancestors					
		Genealogical			Genetic		
Generation (*n*)	Birth year	Mean of expectation	Standard deviation of expectation	Mean of standard deviation	Mean of expectation	Standard deviation of expectation	Mean of standard deviation
0	1610–1615	0.14	0.07	0.54	0.01	0.00	0.09
1	1635–1640	4.25	1.99	3.41	0.41	0.19	0.71
2	1660–1665	14.27	6.04	7.28	2.45	1.03	1.93
3	1685–1690	52.70	19.95	20.47	15.33	5.80	6.91
4	1710–1715	119.90	40.15	42.22	55.60	18.62	20.50
5	1735–1740	96.76	28.37	33.45	65.10	19.09	23.12
6	1760–1765	16.18	4.14	6.60	13.94	3.57	5.88
7	1785–1790	4.10	2.71	2.50	3.97	2.62	2.45
8	1810–1815	2.31	1.57	1.70	2.31	1.57	1.70
9	1835–1840	1.31	0.91	1.20	1.31	0.91	1.19
10	1860–1865	0.94	0.39	0.99	0.94	0.39	0.99
11	1885–1890	0.53	0.23	0.72	0.53	0.23	0.72
12	1910–1915	0.30	0.14	0.53	0.30	0.14	0.53
13	1935–1940	0.17	0.08	0.39	0.17	0.08	0.39
Total	—	313.86	98.58	102.62	162.37	46.72	52.66

		Number of European ancestors					
		Genealogical			Genetic		
Generation (*n*)	Birth year	Mean of expectation	Standard deviation of expectation	Mean of standard deviation	Mean of expectation	Standard deviation of expectation	Mean of standard deviation
0	1610–1615	—	—	—	—	—	—
1	1635–1640	0.32	0.14	0.61	0.03	0.01	0.18
2	1660–1665	1.28	0.61	1.31	0.22	0.10	0.48
3	1685–1690	4.98	2.52	3.10	1.45	0.73	1.36
4	1710–1715	12.98	7.07	6.50	6.02	3.28	3.53
5	1735–1740	16.01	9.44	7.80	10.77	6.35	5.60
6	1760–1765	10.67	6.85	5.58	9.19	5.90	4.96
7	1785–1790	2.38	1.84	1.84	2.30	1.78	1.81
8	1810–1815	1.33	1.04	1.27	1.33	1.04	1.27
9	1835–1840	0.75	0.60	0.90	0.75	0.60	0.90
10	1860–1865	0.19	0.12	0.44	0.19	0.12	0.44
11	1885–1890	0.10	0.06	0.32	0.10	0.06	0.32
12	1910–1915	0.06	0.03	0.24	0.06	0.03	0.24
13	1935–1940	0.03	0.02	0.18	0.03	0.02	0.18
Total	—	51.08	24.32	18.68	32.44	14.31	12.18

Values are calculated as in Table 1. The mean of the expectation for African genealogical ancestors is obtained by averaging values of Eq. 10 across accepted parameter sets (Eq. 15 for genetic ancestors); the standard deviation of the expectation takes the standard deviation of those values. The mean of the standard deviation for African genealogical ancestors is obtained as the mean of Eq. 11 across accepted parameter sets (Eq. 16 for genetic ancestors). For the total, the mean of the expectation of the sum of the African genealogical ancestors is calculated by averaging values of Eq. 12 across accepted parameter sets (Eq. 17 for genetic ancestors); the standard deviation of the expectation takes the standard deviation of those values. The mean of the standard deviation for the total African genealogical ancestors is obtained as the mean of Eq. 13 across accepted parameter sets (Eq. 18 for genetic ancestors). Corresponding quantities for European ancestors are calculated by replacing each $s_{1,g-k}$ with $s_{2,g-k}$. The values of the total means for the expectation and standard deviation of African and European genealogical ancestors are those that appear in Table 3 of Mooney *et al.* (2023). The table shows the generationwise values plotted in Fig. 7 for the means and standard deviations of the expectation across the accepted parameter sets.

By a similar computation, Fig. 5b provides the generationwise expected numbers of African-American genetic ancestors, comparing them to corresponding numbers of genealogical ancestors. The expected total number of African-American genealogical ancestors, summing from generations 0 to 13, is 363, and the expected total for genetic ancestors is 294 (Tables 1 and 3).

In Fig. 7, the peak expected number of African genealogical ancestors appears in generation 4 (1735–1740). However, the corresponding peak for genetic ancestors occurs in generation 5. The difference occurs because the peak for African genealogical ancestors occurs far enough back in time that the probability of genetic ancestry for those genealogical ancestors is well below 1 ($p_{10}=p_{14-4}\approx 0.4637$ by Eq. 1); the number of genetic ancestors among the smaller number of generation-5 genealogical ancestors is greater than among the larger number of generation-4 genealogical ancestors.

For Europeans, the peak of genealogical ancestors occurs later than for Africans, in generation 5 (1760–1765). In that later generation, the fraction of genealogical ancestors who are also genetic ancestors is greater than in generation 4 ($p_{9}=p_{14-5}\approx 0.6728$ by Eq. 1). Because the peak in genealogical ancestors occurs later for Europeans, the fraction of all European genealogical ancestors who are genetic ancestors ($\frac{32}{51}\approx 0.63$) exceeds the corresponding fraction for Africans ($\frac{162}{314}\approx 0.52$).

**Fig. 8.**
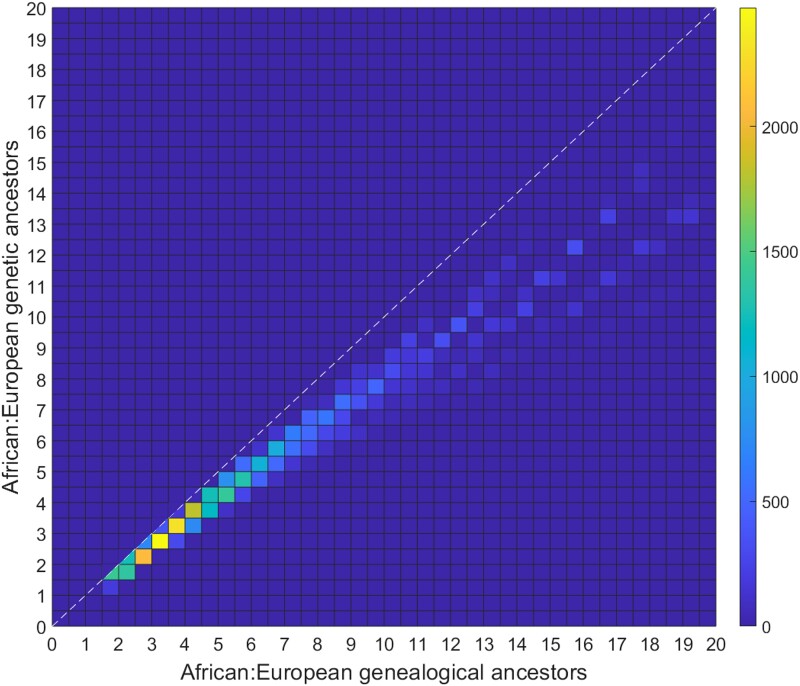
Ratios of the number of African ancestors to the number of European ancestors. The *x*-axis shows the ratio for genealogical ancestors, and the *y*-axis shows the ratio for genetic ancestors. For each of 45,189 accepted parameter sets, we calculated $\left((\sum_{n=0}^{13}E[U_{14-n}])/(\sum_{n=0}^{13}E[U_{14-n}^{\prime }]\right)$, $\left(\sum_{n=0}^{13}E[Y_{14-n}])/(\sum_{n=0}^{13}E[Y_{14-n}^{\prime }])\right)$, visualizing the ordered pair of ratios in a density plot. The 89% of the pairs (40,201) that have both ratios below 20 are presented in the plot, with the color of a $\frac{1}{2}\times \frac{1}{2}$ square representing the number of pairs located in that square. The mean ratios across all accepted parameter sets are (9.99,7.07), and the standard deviations are (11.87,6.32), with covariance 73.56. For the 89% of points shown, the mean ratios are (6.74,5.36), with standard deviations (4.18,2.96) and covariance 12.15. The y=x line is shown for comparison. Among the accepted parameter sets, the ratio we observed for genetic ancestors was always smaller than the ratio for genealogical ancestors; hence, for squares along the diagonal, only the lower triangle is colored. Note that although a smaller value for the ratio of genetic ancestors compared to the ratio of genealogical ancestors was always observed, such a relationship need not hold in principle.

This observation can be illustrated in a computation shown in Fig. 8, which compares the ratio of African and European genetic ancestors to the ratio of African and European genealogical ancestors across accepted parameter sets. The African:European ratio of genetic ancestors is consistently lower than the African:European ratio of genealogical ancestors. The comparative recency of the European genealogical ancestors—and the resulting increased probability of genetic ancestry for those genealogical ancestors—produces a greater value for the fraction of all genetic ancestors who are European compared to the fraction of all genealogical ancestors who are European.

In Fig. 5b, the peak number of African-American genealogical ancestors appears still later than the peaks for African and European genealogical ancestors, in generation 6 (1785–1790). In that generation, the fraction of genealogical ancestors who are also genetic ancestors is $p_{8}=p_{14-6}\approx 0.8615$ (by Eq. 1). Hence, the fraction of African-American genealogical ancestors who are also genetic ancestors ($\frac{294}{363}\approx 0.81$) exceeds corresponding fractions for Africans and Europeans.

## Discussion

We have developed an approach to counting genetic ancestors of an admixed individual, estimating the number of genetic ancestors who contributed directly to the admixed population and the number of genetic ancestors belonging to the admixed population itself. The approach proceeds by recursively treating the number of such ancestors in a given generation as a random variable that is binomially distributed based on a corresponding random variable for the subsequent generation. We used an admixture model together with a model of African-American demographic history to estimate that a random African-American born between 1960 and 1965 has an estimated mean of 162 for the number of African genetic ancestors (standard deviation 47) and 32 for the number of European genetic ancestors (standard deviation 14) who contributed to the African-American population directly from the source populations, and 294 total African-American genetic ancestors (standard deviation 70).

### Genetic and genealogical ancestors

In population-genetic studies of genetically admixed populations, genetic ancestry that traces to the source populations has generally been analyzed by evaluation of estimated admixture fractions in members of an admixed population. The statistical models used for this estimation consider admixture in terms of the fractions of genomes contributed rather than via contributions of specific ancestors. With the increasing use of these genomic contributions to report information to individuals about their own genealogies, the meaning of concepts of genetic ancestry and admixture—and their estimates—have been increasingly queried (Weiss and Long 2009; Lawson *et al.* 2018; Mathieson and Scally 2020). Our use of mechanistic admixture models enables new perspectives on the interpretation of genetic admixture and ancestry estimates, seeking to describe the timing at which the ancestors entered pedigrees of individuals and to count genetic ancestors across the length of the admixture process.

The number of genetic ancestors is bounded above by the number of genealogical ancestors, as each genetic ancestor must also be a genealogical ancestor. Both for genealogical and for genetic ancestors, the number of ancestors in a given generation is binomially distributed based on the number of genealogical ancestors in the subsequent generation (Eqs. 3, 10, 15, 20). The difference between the distributions of genealogical and genetic ancestors is in the binomial probability of success. For genealogical ancestors, the distribution depends only on parameters of the admixture process (Eqs. 3, 10), whereas for genetic ancestors, it depends also on a genetic ancestry probability for a genealogical ancestor separated from a descendant by a specified number of generations (Eqs. 15, 20). Depending on the features of the admixture process, the number of genetic ancestors from a source population can be close to the number of genealogical ancestors, or far smaller (Fig. 4).

The evaluation of genetic ancestors extends the mechanistic admixture model of Mooney *et al.* (2023). From a mathematical perspective, the focus on genealogical ancestors by Mooney *et al.* (2023) proceeded by adding a well-placed factor of 2 to the work of Verdu and Rosenberg (2011), converting a genomic fraction in a single-generation recursion into a genealogical ancestor count. The mathematical extension here is substantial, incorporating into the admixture model not only the factor of 2 but also the time-varying probability that a genealogical ancestor is a genetic ancestor.

Viewed from the perspective of the recombination-based genetic ancestry model of Coop (2013), our approach extends the analysis of genetic ancestors by separating them across source populations. If we were to follow Coop (2013) and consider all populations together as one, then Eq. 3 would reduce to $E[X_{k}]=2^{k}$, and our count of the random number of genetic ancestors in generation *k* would reduce Eq. 20 to $X_{k}^{*}∼\mathrm{Bin}(2^{k},p_{k})$. In other words, with no ancestry proportion considered—or alternatively, with all genealogical ancestors treated as members of the admixed population—the number of genealogical ancestors in generation *k* is $2^{k}$, and the probability that a genealogical ancestor is tabulated as a genetic ancestor depends only on the genetic ancestry probability $p_{k}$. The expectation of this random variable gives the Coop (2013) calculation of the expected number of genetic ancestors in generation *k*, $E[X_{k}^{*}]=2^{k}p_{k}$ (Eq. 1, Fig. 3).

### African-American demographic history

With the Mooney *et al.* (2023) 14-generation model of African-American demographic history, we examined the expected numbers of genetic ancestors from Africa, Europe, and the African-American population itself, for random African-Americans born 1960–1965. We found for the mean numbers of genetic ancestors 162 Africans and 32 Europeans (Fig. 7, Table 3), smaller than the corresponding numbers of genealogical ancestors, 314 Africans and 51 Europeans (Mooney *et al.* 2023). Tabulating ancestors within the African-American population itself, the expected numbers of genealogical and genetic ancestors are 363 and 294, respectively (Fig. 5b, Table 1).

The peak number of genealogical ancestors occurs in generation 4 for Africans (1710–1715), generation 5 for Europeans (1735–1740), and generation 6 for African-Americans (1760–1765, Tables 1 and 4). Tracing genealogical ancestors back in time, noting that the total number of genealogical ancestors doubles in each generation, we find that the proportion of African-Americans among genealogical ancestors is greatest in generation 13, decreasing back in time (Fig. 6a, Table 2). The highest proportion occurs for Africans in generation 5 and for Europeans in generation 6. Eventually, African and European genealogical ancestors are reached who are parents solely of Africans or of Europeans; the proportions of these Africans and Europeans increase back in time until all genealogical ancestors are in these categories, in an approximate ratio of 79% Africans to 21% Europeans (Table 2). These quantities, which estimate fractions of all genealogical ancestors tracing to Africans and Europeans, lie in the range of permissible mean empirical genomic ancestry coefficients (Mooney *et al.* 2023).

For genetic ancestors, the contribution to African genetic ancestry is greatest for generations 4 and 5; the European genetic ancestry is highest in generations 5 and 6 (Fig. 6b). The peak number of genetic ancestors occurs in generation 5 for Europeans and generation 6 for African-Americans, matching corresponding peaks for genealogical ancestors (Tables 1 and 4). However, the peak for African genetic ancestors occurs in generation 5, one generation later than for African genealogical ancestors (Table 4). Many African genealogical ancestors are far enough back in time that many of them are not genetic ancestors—so that the peak for genetic ancestors occurs later for genealogical ancestors. The fact that African genealogical ancestors occur on average farther in the past than European genealogical ancestors means that the 314:51 ratio of the mean numbers of African and European genealogical ancestors is smaller than the 162:32 ratio of the mean numbers of African and European genetic ancestors (Fig. 8), as a larger fraction of the African genealogical ancestors have been lost as genetic ancestors. In effect, the fact that the European genealogical ancestors are later on average than the African genealogical ancestors has the result that the probability that a European genealogical ancestor is also a genetic ancestor exceeds the corresponding probability for Africans.

An interesting difference occurs between the peak of the African ancestor counts and the subsequent peak of the Transatlantic Slave Trade. The fraction of Africans transported by 1760 is about half of the total (Hacker 2020, Table 1); however, the comparable fraction of African genealogical ancestors, individuals born in generation 5 (born 1735–1740, reproductive age at 1760) or earlier, is 92% (Table 4). Hence, although the many transported Africans born in generations 6 and 7 certainly contributed in great numbers to the African-American population, a typical pedigree likely contains multiple lines that trace to the earlier enslaved migrants of generations 5 and earlier. In other words, by the time of the birth of generations 6 (1760–1765) and 7 (1785–1790), the African-American population was large enough that among all genealogical lines of a person born 1960–1965, many trace to genealogical ancestors who were already resident in the African-American population at the time of those generations. Indeed, for generation 6 onward and even for generation 5, African-Americans are a nontrivial fraction of the genealogical ancestors of a modern person (Fig. 6), from ∼38% in generation 6 up to ∼90% in generation 13 (Table 2). The other major component in generation 6 onward is African genealogical ancestors who did not contribute directly to the African-American population. These Africans are the genealogical ancestors of Africans newly contributing to the African-American population. The substantial fraction for this category results from the accumulation of many African genealogical ancestors who contributed to pedigrees in generations later than generation 5.

### Limitations and extensions

As our approach follows the assumptions of Mooney *et al.* (2023), it is subject to many of the same limitations. For example, we do not consider a Native American component of admixture in African-Americans. Our treatment of a “random African-American” born in the 1960–1965 window does not take into consideration regional variation across the African-American population in admixture processes or other demographic phenomena. We also disregard the possibility that the same genealogical ancestor might occur in multiple positions in a pedigree, so that our count of the number of ancestors might double-count some individuals; the time over which this assumption is sensible is the period in which the number of genealogical ancestors in a pedigree is small in relation to the pool of potential ancestors. Our discretization of the generations oversimplifies the demographic history, as does our 3-epoch model, though this model does accord with the perspective of one of the most comprehensive empirical analyses of African-American genetic admixture (Baharian *et al.* 2016). Another limitation is that our model in principle allows an unlikely scenario in which the 2 parents of an African-American are 2 Europeans. We also do not consider distinct ancestry parameters for males and females. Each of these limitations is shared between the assessment of genealogical ancestors by Mooney *et al.* (2023) and our analysis of genetic ancestors here. As is discussed by (Mooney *et al.* 2023, p. 13), each is possible to address by extensions and modifications of the model, potentially leading to further understanding of both genealogical and genetic ancestors.

Additional limitations not shared in the work of Mooney *et al.* (2023), which focused solely on genealogical ancestors, arise from the use of the Coop (2013) model to evaluate the probability that a genealogical ancestor is a genetic ancestor. This approach does not account for recombination phenomena such as recombination-rate variation across the genome, gene conversion, the particular sizes of chromosomes, crossover interference that perturbs the Poisson distribution assumed for the number of new genomic segments each generation, differing male and female recombination rates, or the X chromosome. With its simple treatment of the recombination process, the Coop (2013) model ignores many complexities that affect the probability that some segment from a genealogical ancestor might be retained in a descendant. Although extensions to accommodate such phenomena could be developed, in a single simple equation (Eq. 1), the Coop (2013) recombination model does capture the basic phenomenon—as explained by Donnelly (1983)—that as the time between ancestor and descendant increases, the probability that the descendant retains a segment from the ancestor decreases (Fig. 3), and a steep drop in probability occurs when the separation increases from 7–8 generations (Robert Burns and descendants born 1960–1965) to 15–16 generations (descendants of William Shakespeare).

Our empirical focus has been on an example from human populations, but the model can be applied more generally to diploid species in which mechanistic admixture models and recombination models can be specified. To take one example, Armstrong *et al.* (2023) have studied genetic variation in captive tigers, a population formed through admixture of wild source populations from several different parts of Asia. Armstrong *et al.* (2023) have estimated genomic proportions that trace to the various source populations. With a generalization to permit more than 2 sources, our model can assist in understanding the properties of the genetic ancestors that have given rise to typical individual captive tigers.

### Conclusions

Further study of a mechanistic admixture model has deepened the analysis of the number of genealogical ancestors who contribute from a source population to an admixed pedigree, and it has also introduced an approach to evaluating the number of contributing genetic ancestors. For African-Americans, the distinction between genealogical and genetic ancestors suggests that although the number of African genealogical ancestors in a pedigree greatly exceeds the number of European genealogical ancestors, because the African genealogical ancestors are on average earlier in time than the European genealogical ancestors, the number of African *genetic* ancestors does not exceed the number of European genetic ancestors by as great a margin. More generally, the calculations contribute to understanding the relationship between an admixed population’s demographic history, its ancestral individuals who have given rise to the modern population, and the genomes of its current members.

## Supplementary Material

iyae011_Supplementary_Data

## Data Availability

The 45,189 sets of accepted parameter values $\left(s_{1,0},h_{0},s_{2,0}\right)$, $\left(s_{1,1},h_{1},s_{2,1}),\ldots ,(s_{1,13},h_{13},s_{2,13}\right)$ from Mooney *et al.* (2023), on which the analysis of the African-American population is based, are available in Supplementary File 1. Supplemental material is available at GENETICS online.

## Appendix A: Proofs of Eqs. 3, 4 and 6

We prove Eq. 3, describing $E[X_{k}]$, by induction. For k=0,

$$X_{k}=1=2^{0}\prod_{i=1}^{0}h_{g-i}.$$

We assume that for k−1,

$$E[X_{k-1}]=2^{k-1}\prod_{i=1}^{k-1}h_{g-i}.$$

Using the inductive hypothesis and the fact that $X_{k}∼\mathrm{Bin}(2X_{k-1},h_{g-k})$ for 1≤k≤g, we obtain

$$\begin{array}{rl} E[X_{k}] & =E[E[X_{k}|X_{k-1}]]=2h_{g-k}E[X_{k-1}]\\ & =2h_{g-k}2^{k-1}\prod_{i=1}^{k-1}h_{g-i}=2^{k}\prod_{i=1}^{k}h_{g-i}. \end{array}$$

Next, we prove Eq. 4, again by induction. For k=0, $X_{0}=1$ has variance 0; Eq. 4 holds trivially, as it is an empty sum. For k=1, $X_{1}∼\mathrm{Bin}(2,h_{g-1})$, and therefore,

$$\begin{array}{rl} \mathrm{Var}[X_{1}] & =2h_{g-1}(1-h_{g-1})\\ & =\sum_{i=1}^{1}2^{1-1+i}[1-h_{g-(1+1)+i}]\\ & \;\times \left[\left(\prod_{\;j=g-(1+1)+i}^{g-1}h_{j}\right)\left(\prod_{\ell =g-1}^{g-(1+2)+i}h_{\ell }^{2}\right)\right]. \end{array}$$

We assume that for k−1,

$$\begin{array}{rl} \mathrm{Var}[X_{k-1}] & =\sum_{i=1}^{k-1}2^{k-2+i}(1-h_{g-k+i})\\ & \;\times \left[\left(\prod_{\;j=g-k+i}^{g-1}h_{j}\right)\left(\prod_{\ell =g-(k-1)}^{g-(k+1)+i}h_{\ell }^{2}\right)\right]. \end{array}$$

We use the law of total variance with Eq. 3 and the inductive hypothesis. We have

$$\begin{array}{rl} \mathrm{Var}[X_{k}] & =E[\mathrm{Var}[X_{k}|X_{k-1}]]+\mathrm{Var}[E[X_{k}|X_{k-1}]]\\ & =E[2h_{g-k}(1-h_{g-k})X_{k-1}]+\mathrm{Var}[2h_{g-k}X_{k-1}]\\ & =2h_{g-k}(1-h_{g-k})2^{k-1}\left(\prod_{i=1}^{k-1}h_{g-i}\right)\\ & \;+(2h_{g-k}{)}^{2}\sum_{i=1}^{k-1}2^{k-2+i}(1-h_{g-k+i})\\ & \;\times \left[\left(\prod_{\;j=g-k+i}^{g-1}h_{j}\right)\left(\prod_{\ell =g-(k-1)}^{g-(k+1)+i}h_{\ell }^{2}\right)\right]\\ & =2^{k}(1-h_{g-k})\left(\prod_{\;j=g-k}^{g-1}h_{j}\right)\\ & \;+\sum_{i=2}^{k}2^{2+k-2+i-1}(1-h_{g-k+i-1})\\ & \;\times \left[\left(\prod_{\;j=g-k+i-1}^{g-1}h_{j}\right)\left(\prod_{\ell =g-k}^{g-(k+1)+i-1}h_{\ell }^{2}\right)\right]\\ & =\sum_{i=1}^{k}2^{k-1+i}[1-h_{g-(k+1)+i}]\\ & \;\times \left[\left(\prod_{\;j=g-(k+1)+i}^{g-1}h_{j}\right)\left(\prod_{\ell =g-k}^{g-(k+2)+i}h_{\ell }^{2}\right)\right]. \end{array}$$

Finally, we prove Eq. 6. First, we prove that if 0≤m<n≤g, then

$$\mathrm{Cov}[X_{n},X_{m}]=2^{n-m}\prod_{i=1}^{n-m}h_{g-(m+i)}\mathrm{Var}[X_{m}].$$

Fixing *m* with 0≤m≤g−1, we proceed by induction on *n*. For n=m+1, we have

$$\begin{array}{rl} \mathrm{Cov}[X_{m+1},X_{m}] & =E[X_{m+1}X_{m}]-E[X_{m+1}]E[X_{m}]\\ & =E[E[X_{m+1}X_{m}\;|\;X_{m}]]\\ & \;-2h_{g-(m+1)}E[X_{m}]E[X_{m}]\\ & =E[2h_{g-(m+1)}X_{m}^{2}]-2h_{g-(m+1)}E[X_{m}]E[X_{m}]\\ & =2^{m+1-m}h_{g-(m+1)}\mathrm{Var}[X_{m}]. \end{array}$$

We now assume that for (n,m) with 0≤m<n≤g and n≥m+2,

$$\mathrm{Cov}[X_{n-1}X_{m}]=2^{n-1-m}\prod_{i=1}^{n-1-m}h_{g-(m+i)}\mathrm{Var}[X_{m}].$$

Then

$$\begin{array}{rl} \mathrm{Cov}[X_{n},X_{m}] & =E[X_{n}X_{m}]-E[X_{n}]E[X_{m}]\\ & =E[E[X_{n}X_{m}\;|\;X_{m},X_{n-1}]]\\ & \;-2h_{g-n}E[X_{n-1}]E[X_{m}]\\ & =2h_{g-n}(E[X_{n-1}X_{m}]-E[X_{n-1}]E[X_{m}])\\ & =2h_{g-n}2^{n-1-m}\prod_{i=1}^{n-1-m}h_{g-(m+i)}\mathrm{Var}[X_{m}]\\ & =2^{n-m}\prod_{i=1}^{n-m}h_{g-(m+i)}\mathrm{Var}[X_{m}]. \end{array}$$

Having obtained the covariance $\mathrm{Cov}[X_{n},X_{m}]$, we conclude

$$\begin{array}{rl} \mathrm{Var}\left[\sum_{k=1}^{g}X_{k}\right] & =\sum_{k=1}^{g}\mathrm{Var}[X_{k}]\\ & \;+2\sum_{m=1}^{g-1}\sum_{n=m+1}^{g}\mathrm{Cov}[X_{n},X_{m}]\\ & =\sum_{k=1}^{g}\mathrm{Var}[X_{k}]\\ & \;+\sum_{m=1}^{g-1}\sum_{n=m+1}^{g}2^{n-m+1}\prod_{i=1}^{n-m}h_{g-(m+i)}\mathrm{Var}[X_{m}]. \end{array}$$

## Appendix B: Proofs of Eqs. 13 and 18

We prove Eq. 13, starting with the law of total variance.

$$\begin{array}{rl} \mathrm{Var}\left[\sum_{k=1}^{g}U_{k}\right] & \;\overset{\left(i\right)}{=}\;\mathrm{Var}\left[E\left[\sum_{k=1}^{g}U_{k}\;|\;X_{0},X_{1},\ldots ,X_{k}\right]\right]\\ & \;+E\left[\mathrm{Var}\left[\sum_{k=1}^{g}U_{k}\;|\;X_{0},X_{1},\ldots ,X_{k}\right]\right]\\ & \;\overset{\left(ii\right)}{=}\;\mathrm{Var}\left[\sum_{k=1}^{g}{\tilde{s}}_{1,g-k}(2X_{k-1}-X_{k})\right]\\ & \;+E\left[\sum_{k=1}^{g}{\tilde{s}}_{1,g-k}(1-{\tilde{s}}_{1,g-k})(2X_{k-1}-X_{k})\right]\\ & \;\overset{\left(iii\right)}{=}\;\mathrm{Var}\left[\sum_{k=1}^{g-1}[2{\tilde{s}}_{1,g-(k+1)}-{\tilde{s}}_{1,g-k}]X_{k}\right]\\ & \;+\sum_{k=1}^{g}{\tilde{s}}_{1,g-k}(1-{\tilde{s}}_{1,g-k})E\left[E[2X_{k-1}-X_{k}\;|\;X_{k-1}]\right]\\ & \;\overset{\left(iv\right)}{=}\;\sum_{k=1}^{g-1}[2{\tilde{s}}_{1,g-(k+1)}-{\tilde{s}}_{1,g-k}{]}^{2}\;\mathrm{Var}[X_{k}]\\ & \;+\sum_{m=1}^{g-2}\sum_{n=m+1}^{g-1}2^{n-m+1}[2{\tilde{s}}_{1,g-(m+1)}-{\tilde{s}}_{1,g-m}]\\ & \;\times [2{\tilde{s}}_{1,g-(n+1)}-{\tilde{s}}_{1,g-n}]\prod_{i=1}^{n-m}h_{g-(m+i)}\;\mathrm{Var}[X_{m}]\\ & \;+\sum_{k=1}^{g}{\tilde{s}}_{1,g-k}(1-{\tilde{s}}_{1,g-k})(1-h_{g-k})(2E[X_{k-1}])\\ & \;\overset{\left(v\right)}{=}\;\sum_{k=1}^{g-1}[2{\tilde{s}}_{1,g-(k+1)}-{\tilde{s}}_{1,g-k}{]}^{2}\;\mathrm{Var}[X_{k}]\\ & \;+\sum_{m=1}^{g-2}\sum_{n=m+1}^{g-1}2^{n-m+1}[2{\tilde{s}}_{1,g-(m+1)}-{\tilde{s}}_{1,g-m}]\\ & \;\times [2{\tilde{s}}_{1,g-(n+1)}-{\tilde{s}}_{1,g-n}]\prod_{i=1}^{n-m}h_{g-(m+i)}\;\mathrm{Var}[X_{m}]\\ & \;+\sum_{k=0}^{g-1}2s_{1,g-(k+1)}[1-{\tilde{s}}_{1,g-(k+1)}]E[X_{k}]. \end{array}$$

For line (ii), given $X_{0},\ldots ,X_{k-1},X_{k}$, $U_{k}$ depends only on $X_{k-1}$ and $X_{k}$. Among the genealogical ancestors in step *k* of the descendant from step 0, $2X_{k-1}$ are parents of admixed individuals from step k−1, and $X_{k}$ are admixed individuals in step *k*; $2X_{k-1}-X_{k}$ reach a source population in step *k*, with binomial probabilities $s_{1,g-k}/(s_{1,g-k}+s_{2,g-k})=s_{1,g-k}/(1-h_{g-k})={\tilde{s}}_{1,g-k}$ for source 1 and $s_{2,g-k}/(s_{1,g-k}+s_{2,g-k})=s_{2,g-k}/(1-h_{g-k})={\tilde{s}}_{2,g-k}$ for source 2, respectively. In other words, $U_{k}\;|\;X_{k-1},X_{k}∼\mathrm{Bin}(2X_{k-1}-X_{k},{\tilde{s}}_{1,g-k})$.

For line (iii), in the sum $\sum_{k=1}^{g}{\tilde{s}}_{1,g-k}(2X_{k-1}-X_{k})$, for k=1,2,…,g−1, $X_{0}=1$ and $X_{g}=0$ are constants and have zero variance. We also use the law of total expectation. Line (iv) follows from Eq. 6 and from the binomial distribution of $X_{k}\;|\;X_{k-1}$, so that $E\left[E[2X_{k-1}-X_{k}\;|\;X_{k-1}]\right]=2E[X_{k-1}]-2h_{g-k}E[X_{k-1}]=(1-h_{g-k})(2E[X_{k-1}])$. Finally, for (v), we simplify ${\tilde{s}}_{1,g-k}(1-h_{g-k})=s_{1,g-k}$.

Similarly, we also use the law of total variance to prove Eq. 18:

$$\begin{array}{rl} \mathrm{Var}\left[\sum_{k=1}^{g}Y_{k}\right] & =\mathrm{Var}\left[E\left[\sum_{k=1}^{g}Y_{k}\;|\;X_{0},X_{1},\ldots ,X_{k}\right]\right]\\ & \;+E\left[\mathrm{Var}\left[\sum_{k=1}^{g}Y_{k}\;|\;X_{0},X_{1},\ldots ,X_{k}\right]\right]. \end{array}$$

The proof is entirely analogous, except that ${\tilde{s}}_{1,g-k}p_{k}$ appears in place of ${\tilde{s}}_{1,g-k}$.

## Appendix C: Proof of Eq. 35

We prove inequalities concerning $\;p_{k}/\;p_{k-1}$: (1) $\;p_{k}/\;p_{k-1}<1$ for k≥2; (2) $\;p_{k}/\;p_{k-1}>\frac{1}{2}$ for k≥2.

1. By Eq. 1, $\;p_{k}/\;p_{k-1}=\left[1-e^{-a(k)}\right]/\left[1-e^{-b(k)}\right]$ for k≥3, where $a(k)=(33k-11)/2^{k-1}$ and $b(k)=(33k-44)/2^{k-2}$. For k≥3, 0<a(k)<b(k), and hence, $1-e^{-a(k)}<1-e^{-b(k)}$ and $\;p_{k}/\;p_{k-1}<1$. For k=2, $\;p_{k}/\;p_{k-1}<1$ as $p_{k}<1$ by Eq. 1 and $p_{k-1}=1$.
2. For k=2, $p_{k}/p_{k-1}=p_{2}=1-e^{-55/2}>\frac{1}{2}$. For k≥3, we rearrange Eq. 1 to find that the inequality $p_{k}/p_{k-1}>\frac{1}{2}$ is equivalent to
  (C1) $$e^{\frac{66}{2^{k-1}}}e^{\frac{33k-77}{2^{k-1}}}+e^{-\frac{33k-77}{2^{k-1}}}>2.$$
  The inequality $e^{x}+e^{-x}\geq 2$ holds for all *x*, as it is equivalent to coshx≥1. Hence, for c>1, $ce^{x}+e^{-x}>e^{x}+e^{-x}\geq 2$. We see that Eq. C1 then follows, with
  $$\left(e^{\frac{66}{2^{k-1}}},\frac{33k-77}{2^{k-1}}\right)$$
  in place of (c,x). As Eq. C1 holds, we conclude $p_{k}/p_{k-1}>\frac{1}{2}$.
